# C-terminal interleukin 1 alpha (IL-1α) overexpression drives EMT and a vulnerability to ferroptosis in HNSCC

**DOI:** 10.1016/j.redox.2026.104172

**Published:** 2026-04-16

**Authors:** Ishrat Nourin Khan, Krishna Awasthi, Ziyu Wang, Nafis Md Irfan, Jay Saepoo, Joan N. Whittier, Nurgul Koyuncu, Md Roman Mogal, M.M. Hasibuzzaman, Shujie Yang, Michael Petronek, Andrean L. Simons

**Affiliations:** a Interdisciplinary Graduate Program in Human Toxicology, University of Iowa, Iowa City, IA, USA; b Iowa City Veterans Affairs Health Care System, Iowa City, IA, USA; c Free Radical and Radiation Biology Program, Department of Radiation Oncology, University of Iowa Hospitals and Clinics, Iowa City, IA, USA; d Holden Comprehensive Cancer Center, University of Iowa Hospitals and Clinics, Iowa City, IA, USA; e Department of Pathology, University of Iowa Hospitals and Clinics, Iowa City, IA, USA

**Keywords:** HNSCC, IL-1α, Ferroptosis, EMT, EGFR

## Abstract

High tumor expression of the cytokine interleukin-1 alpha (IL-1α) is associated with an aggressive tumor phenotype and poor clinical outcomes in head and neck squamous cell carcinoma (HNSCC). However, the mechanism behind IL-1α-induced tumor aggressiveness is unclear. The goal of this work is to investigate the biological consequences of increased IL-1α in HNSCC cells.

Three *IL1A* constructs (Full-length, N-terminal, and C-terminal [CT]) were transduced into Cal27 HNSCC cells and validated for IL-1α expression. Differences in cell survival, metabolic profiling, epithelial-to-mesenchymal transition (EMT), oxidative stress parameters and response to therapy were compared among the parental and IL-1α overexpressing cell lines.

Overexpression of CT IL-1α (but not the other constructs) led to increased cell proliferation, membrane fluidity, hydroperoxide production, intracellular iron and lipid peroxidation, EMT changes, and a shift toward glutamate utilization compared to control cell lines. Finally, CT IL-1α cells (but not the other constructs) were highly sensitive to the ferroptosis inducer RSL3.

Together this work suggests that increased tumor IL-1α expression triggers a shift toward an oxidative and ferroptotic environment, leading to an aggressive tumor phenotype and drug resistance; but also reveals a unique vulnerability to agents that induce ferroptosis.

## Highlights

• Metabolic changes toward glutamate metabolism accompany increased tumor IL-1α expression.
• Sustained tumor IL-1α expression drives EMT and resistance to EGFR tyrosine kinase inhibitors.
• Increased tumor IL-1α drives oxidative stress and iron-dependent lipid peroxidation.
• High tumor IL-1α expression promotes sensitivity to RSL3.

## Graphical abstract

**Figure:**
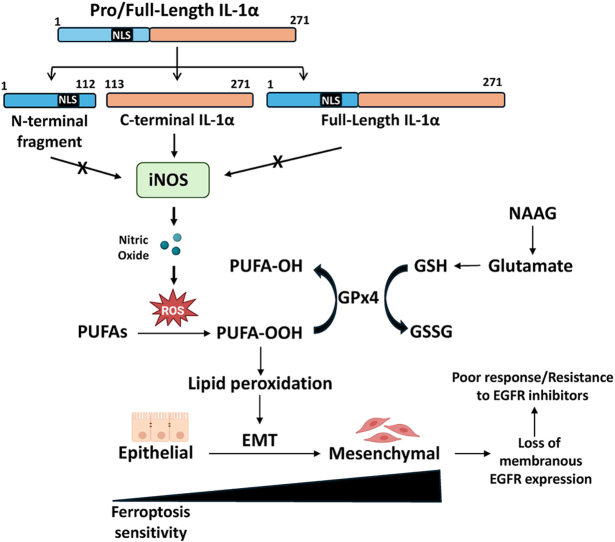

## 1 Introduction

Most head and neck squamous cell carcinoma (HNSCC) patients present with advanced locoregional disease at diagnosis and remain at a high risk of developing secondary tumors despite achieving local control with radiotherapy and systemic chemotherapy [1,2]. The primary disease-related mortality in HNSCC patients is due to locoregional failure [[1], [2], [3]], and these failures arise from biologically aggressive tumor cell subpopulations that promote immune evasion, drug resistance and radio-resistance. There have been major advances in genomic profiling for HNSCC patients [4,5], however predictive biomarkers for treatment response remain limited. Therefore, there is a strong need to identify functional biomarkers that capture tumor behavior and guide precision treatment strategies for this disease.

Unlike other solid tumors, where actionable mutations or robust immune signatures can guide therapy selection (e.g. non-small cell lung cancer, melanoma, breast cancer, colorectal cancer), most HNSCC patients are treated without reliable biomarkers to predict response, resistance, or toxicity. Human papilloma virus (HPV)-positivity, which is prevalent in oropharyngeal squamous cell carcinomas (OPSCCs), provides important prognostic information and is a general indicator of radio-sensitivity [6], but offers limited predictive value for therapeutic decision-making [7,8]. Epidermal growth factor receptor (EGFR) expression is a prognostic factor for tumor recurrence in HNSCC patients treated with surgery and postoperative radiotherapy [9], but does not predict response to EGFR inhibitors [9,10]. PD-L1 expression is an important predictor of response to anti-PD1/PDL1 agents [11] but is limited by intratumoral and intertumoral heterogeneity [12], dynamic tumor expression over time [13], assay and scoring variability [14], and response rates to immunotherapy are modest (15-20%) even in PD-L1 high tumors [15]. As a result, more work should be carried out to identify effective predictive biomarkers to accurately predict therapeutic outcomes for HNSCC.

Our previous work and others have shown that high tumor expression of interleukin-1 alpha (IL-1α) is associated with aggressive tumor behavior and poor clinical outcomes across multiple cancer types including HNSCC [[16], [17], [18], [19], [20]]. As a dual function cytokine, IL-1α plays a central role in immune and inflammatory responses. IL-1α binds to IL-1 receptor type I (IL-1R1) which forms a complex with the IL-1 receptor accessory protein (IL-1RAP) leading to the recruitment of myeloid differentiation primary response gene 88 (MyD88), IL-1 receptor-associated kinases (IRAKs) and tumor necrosis factor receptor-associated factor 6 (TRAF6) [21]. This signaling triggers the activation of the nuclear factor-kappa B (NFκB) and mitogen-activated protein kinase (MAPK) pathways resulting in the expression of IL-1 target genes. Tumor-specific IL-1 signaling activates pathways leading to the expression of numerous pro-inflammatory cytokines which promote and sustain tumor survival contributing to poor drug response [22]. IL-1α has been previously associated with antiapoptotic signals [23], angiogenesis [24], migration and metastasis [25], stemness [26] and modulation of the tumor microenvironment (TME) including activation of stromal cells [27] and immunosuppression [28,29]. In HNSCCs, elevated tumor IL-1α gene expression was found to correlate with increased risk of distant metastasis and significantly worse distant metastasis–free survival [16]. Increased IL-1α protein expression was associated with tumor aggressiveness and reduced overall survival [30,31]. Our previous work in oral squamous cell carcinoma (OSCC) has shown that tumor IL-1α protein expression was a strong indicator of tumor recurrence in EGFR-positive OSCC patients [32]. Beyond HNSCC, high IL-1α gene/protein expression has similarly been linked to poor overall survival in renal cell carcinoma [33] and bladder cancer [34], further supporting its adverse prognostic significance.

Despite the prognostic potential of IL-1α expression in HNSCCs, the mechanism behind the ability of IL-1α to increase tumor aggressiveness is not clear. It is well established that IL-1α exists as a full-length 31 kDa protein that can be cleaved into C-terminal 17 kDa IL-1α and a 16 kDa N-terminal fragment [35,36]. Here we overexpressed these three IL-1α constructs (full-length [FL], N-terminal [NT], and C-terminal [CT]) in the Cal27 HNSCC cell line and conducted a series of experiments to understand the biological consequences of increased IL-1α expression. We found that overexpression of the CT IL-1α (but not the other constructs) led to the development of a ferroptotic environment and epithelial to mesenchymal transition (EMT) leading to a more aggressive and therapy-resistant phenotype. This work suggests that high IL-1α expressing tumors may be uniquely sensitive to ferroptosis-inducing agents and lays the foundation for additional work investigating the predictive value of tumor-derived IL-1α.

## 2 Methods and materials

### 2.1 Gene expression database sources

Pan cancer *IL1A* gene expression data was accessed and downloaded through the UALCAN (University of Alabama at Birmingham Cancer Data Analysis Portal) web tool (http://ualcan.path.uab.edu/analysis.html) [37,38], which facilitates user-friendly access to TCGA data for analyses of gene expression profiles and select associated clinical characteristics. *IL1A* expression levels were compared between HNSCC tumor and normal tissue samples. A dataset of gene expression of 604 HNSCC samples (GDC TCGA Head and Neck Cancer (HNSC) along with the corresponding clinical outcomes were downloaded from The Cancer Genome Atlas (TCGA) using Xena Functional Genomics Explorer (https://xenabrowser.net/, University of California, Santa Cruz). Patients were divided into quartiles according to their *IL1A* gene expression levels and labeled as ‘high’ (n = 140) and ‘low’ (n = 142) *IL1A* gene expression. The two gene expression groups were analyzed for differences in overall survival using Kaplan-Meier curves.

### 2.2 Cell lines and reagents

Cal27 cell line was obtained from American Type Culture Collection (ATCC, CRL-2095) and was cultured in high glucose Dulbecco's Modified Eagle's Medium (DMEM) containing 4 mM l-glutamine, 4.5 g/L glucose with 10% Fetal Bovine Serum (FBS) and 0.1% gentamycin solution. Cell cultures were maintained in a 5% CO_2_ incubator at 37 °C and 95% humidity. Parental and IL-1α-overexpressing Cal27 cell lines were stored according to the supplier's instructions and the cell line was subcultured every 3–4 days using trypsin-EDTA. When necessary, the cells were collected with trypsin-EDTA and counted using a coulter counter Z1 series (Beckman Coulter, Brea, CA). Erlotinib, RSL3 (ras-selective lethal 3), and ferrostatin 1 (Fer-1) were purchased from Cayman chemical (MI, USA). Afatinib, osimertinib and silivertinib (BDTX-1535) were from MedchemExpress (NJ, USA).

### 2.3 Lentiviral transduction

Lentiviral expression plasmids encoding full-length human interleukin-1 alpha (*IL1A*; amino acids 1–271) was generated in the pGenLenti backbone by GenScript (NJ, USA) using CloneEZ cloning (WT full-length *IL-1A* [1-271 aa] pGenLenti). Two additional *IL1A* constructs were derived from the full-length *IL1A* pGenLenti plasmid by site-directed mutagenesis to express the N-terminal *IL1A* (aa 1-112) and the C-terminal *IL1A* (aa 113-271). These fragments were generated using *Nhe*I and *Pme*I restriction sites according to vendor specifications. The control pGenLenti empty vector was generated in parallel and used as a negative control. Lentiviral transduction with the plasmids was performed by transfecting Lenti-X™ 293T lentiviral packaging cells (Takara Bio, USA) with 10 μg of plasmid DNA. Transfection was done using Lipofectamine Reagent and Plus Reagent (Invitrogen, Life Technologies, Carlsbad, CA, USA) according to manufacturer's instructions. Supernatant was collected at 48 and 72 h after transfection, filtered with 0.45 μm filters and supplemented with 8 μg/mL polybrene (Invitrogen, Life Technologies, Carlsbad, CA, USA). Serial transductions (48 and 72-h supernatants) were applied to Cal27 cells for 8 h. Pooled stable lines were generated by selecting with puromycin for one week.

### 2.4 Quantitative real-time (reverse transcription) PCR

IL-1α overexpressed Cal27 cells were seeded in 100 mm dishes (2 x 10^6^ cells/dish) and cultured for 48 h before isolating total RNA using RNeasy Plus mini kit (QIAGEN, Hilden, Germany)

as per the manufacturer's protocol. The RNA purity was measured by NanoDrop (Thermo Fisher Scientific, Waltham, MA, USA). One μg of RNA was reverse-transcribed into cDNA using the iScript cDNA Synthesis kit (Bio-Rad Laboratories, Hercules, CA, USA). Target gene expression (Supplementary Table 1) was assessed by qRT-PCR reaction containing iTaq Universal SYBR Green Supermix (Bio-Rad). qRT-PCR was performed using the QuantStudio 3 real-time PCR system (Thermo Fisher Scientific). Target genes were normalized to the housekeeping gene GAPDH and change in gene expression was determined using the delta delta cycle threshold (ΔΔCt) method. Each assay was performed in triplicate and results were presented as mean ± standard error of mean.

### 2.5 ELISA and western blotting

Cell culture media from IL-1α overexpressed Cal27 cells were collected for analyzing the levels of IL-1α, IL-1β, IL-6, IL-8 or IL-1RA using Human Duo Set ELISA kits (R&D Systems, Minneapolis, MN) according to the manufacturer's protocols. Colorimetric analyses were done using a Synergy H1 Hybrid Multi-Mode Reader (BioTek, Winooski, VT). For Western blot, whole cells were lysed in the RIPA lysis buffer (Thermo Fisher) whereas the cytoplasmic and nuclear fractions were extracted using NE-PER™ Nuclear and Cytoplasmic Extraction Reagents (Thermo Fisher). Protein concentration was measured using the Bradford assay, and 20–50 μg of protein per sample was loaded onto a NuPage 4-12% Bis Tris gel (Thermo Fisher). After transfer to nitrocellulose membranes (Invitrogen), separated proteins were probed with selected primary antibodies (listed in Supplementary Table 2), followed by Anti-rabbit IgG HRP-linked antibody or Anti-mouse IgG, HRP-linked antibody (Cell signaling) secondary antibodies (dilution 1:5000). Images were acquired using the ChemiDoc Imaging Systems (BioRad), and signals from 3 biological replicates were quantified by densitometry relative to the housekeeping proteins α-tubulin or GAPDH, using the Image Lab™ Software (Bio-Rad)

### 2.6 Cell proliferation and survival analysis

Cell proliferation was determined using the Coulter counter Z1 series (Beckman). The cells were seeded in 6 well plates (2 x 10^5^ cells/well) and were counted every 24 h for 72 h, and the obtained results were analyzed using the GraphPad prism v 10.6.1 (GraphPad Software, San Diego, CA, USA). Cell viability (MTT) assays were carried out in 96-well plates (Corning) and in each well 5000 cells were plated and harvested overnight. After drug treatment (48h), cell media was removed, and cells were rinsed with 1X PBS then 100 μl MTT (3-(4,5-dimethylthiazol-2-yl)-2,5-diphenyltetrazolium bromide) (Tocris Bioscience, Bristol, UK; Cat. No. 5224) solution (1 mg/mL in clear medium without serum and phenol red) was added to each well and the plates were incubated at 37 °C for 1-4 h. During the incubation, the active enzymes of the viable cells transformed the yellow MTT into purple formazan crystals. The MTT solution was then removed and DMSO was added to each well to dissolve the formazan crystals. The absorbance of the solution was determined at 570 nm by using a Synergy H1 Hybrid Multi-Mode Reader (BioTek, Winooski, VT). For clonogenic assays, a total of 1 × 10^5^ cells were seeded into 60 mm dishes and allowed to grow for 24 h. Cells were then treated with RSL3 at final concentrations of 0.5 μM or 1 μM or with control (DMSO), for 24 h. After treatment cells were collected by trypsinization, and pelleted (1200 rpm for 5 min). Cell pellets were then resuspended in fresh medium, and the total cell population was determined using a Coulter Counter (Beckman). Cells were re-plated in six-well plates in specific dilutions and were allowed to form colonies over 12–14 days in complete medium supplemented with 0.1% gentamycin. At the end of the incubation period, colonies were fixed with 70% ethanol for 5 min and stained with Coomassie blue solution (0.4 w/v in a 1:4:1 mixture of methanol, water and acetic acid). Only colonies consisting of more than 50 cells were counted. Surviving fractions were determined by normalizing the plating efficiencies (Number of colonies observed/Number of cells plated × 100) of treated groups to the corresponding untreated control group.

### 2.7 Apoptosis assays

Cellular apoptosis was evaluated using the Annexin V-FITC Apoptosis Detection Kit (ab14085, Abcam) according to the manufacturer's guidelines. IL-1α-overexpressing Cal27 cells were treated with RAS-selective lethal 3 (RSL3, 1 μM) with or without pretreatment (45 min) with Ferrostatin-1 (Fer-1, 2 μM). DMSO was used as a vehicle control. Cells were the washed twice with PBS and resuspended in 500 μl 1X Annexin V binding buffer followed by incubation with Annexin V-FITC and propidium iodide (PI) for 15 min in the dark at room temperature. Samples were then analyzed in flow cytometry (Attune NxT Acoustic Focusing Cytometer, Thermo Fisher Scientific). Percentage of cells that were viable, in early or late apoptosis, or necrotic were evaluated using Annexin V and P staining. Analysis was done using FlowJo software (v10; FlowJo LLC, Ashland, OR, USA).

### 2.8 Membrane fluidity assay

Membrane fluidity of the IL-1α overexpressed Cal27 cells were evaluated using the membrane fluidity kit (ab189819, Abcam, Cambridge, UK) as per manufacturer's instructions. Cells were incubated for 1 h at 25 °C in the dark with the fluorescent lipid reagent pyrenedecanoic acid (PDA) (5 μM) with 0.08 % Pluronic F127. Cells were then washed with PBS, trypsinized, and centrifuged to collect the stained cell pellets and flow cytometry analysis were performed on an LSR II Flow Cytometer (BD Biosciences). Fluorescence was monitored with 405 nm filter for monomers and 460 nm filter for excimer fluorescence. The ratio of excimer to monomer fluorescence was used to calculate the relative membrane fluidity.

### 2.9 RNA sequencing and metabolomics

Targeted reverse phase (RP) liquid chromatography (LC)-mass spectrometry (MS) metabolite analysis was performed by the Northwest Metabolomics Research Center following published protocols (University of Washington, Seattle, Washington) [39,40] to quantify 372 metabolites in the IL-1α-overexpressing Cal27 cells. A total of 200 metabolites were measured across study set and the relative metabolites intensities were analyzed using MetaboAnalyst 5.0. RNA sequencing of the IL-1α overexpressing cell lines was performed by the Novogene (Sacramento, CA, USA). RNA was extracted from the cells using the RNeasy Plus Mini kit (Qiagen) and mRNA library was prepared using poly A enrichment. Human mRNA Sequencing was performed on an Illumina NovaSeq X Plus platform using 150 bp paired end reads. Data analysis was performed using NovoMagic (Novogene).

### 2.10 *In vivo* tumor formation

Female NU/J mice (4-6 weeks old, Strain #002019; The Jackson Laboratory) were housed in the Animal Care Facility at the University of Iowa. They were handled with aseptic procedures and were allowed to acclimate for at least 7 days before handling. Food and water were readily accessible to the mice. All animal procedures were approved by the Institutional Animal Care and Use Committee (IACUC) at the University of Iowa and adhered to the guidelines set by the National Institutes of Health. The IL-1α overexpressed Cal27 HNSCC cell lines (1 × 10^6^ cells/100 μL PBS) were inoculated subcutaneously into the right flank of each animal. Time to tumor formation was recorded when tumors became palpable (∼3-4 mm in any direction). Tumor measurements (using Vernier calipers) were evaluated, and tumor volumes were calculated using the formula: tumor volume = (length × width2)/2, where length is the longest dimension and width is the dimension perpendicular to the length. Mice were euthanized by CO_2_ gas asphyxiation when the tumor size exceeded 15 mm in any dimension.

### 2.11 Intracellular prooxidant analyses

Intracellular reactive oxygen species (ROS) levels were evaluated by the 5-(and-6)-carboxy-2′,7′-dichlorodihydrofluorescein diacetate (DCFDA/H2DCFDA) Cellular ROS Assay Kit (ab113851, Abcam, Cambridge, UK) according to the manufacturer's guidelines. IL-1α overexpressing Cal27 cells were seeded in 100 mm dishes at a density of 5 x 10^5^ cells/dish and cultured for 48 h. Cells were then tripsinized, washed with PBS resuspended in FACS tube and stained with freshly prepared DCFDA working solution (20 μM) for 30 min at 37 °C and 5% CO_2_ in the dark. Cells were immediately analyzed by flow cytometry (Attune NxT Acoustic Focusing Cytometer, Thermo Fisher Scientific) using excitation/emission settings 488/535 nm. Appropriate unstained control samples were included to establish baseline fluorescence. ROS levels were quantified as the mean fluorescence intensity (MFI, geometric mean) using FlowJo software (v10; FlowJo LLC) in the gated 10,000 live cell population.

Lipid peroxidation was assessed using the BODIPY 581/591 C11 fluorescent probe. Control and drug-treated cells were stained with BODIPY 581/591 C11 (2 μM in PBS + 1% FBS) for 30 min at 37 °C in the dark, pelleted (300×*g*, 5 min), washed twice, and stained with Zombie Violet viability dye (1:200 dilution) for 15 min at room temperature. Cells were washed twice, resuspended in 300 μL of PBS +1% FBS, and analyzed using flow cytometry (Attune NxT Acoustic Focusing Cytometer, Thermo Fisher Scientific)

### 2.12 Intracellular iron analyses

Cytoplasmic Fe2+ levels were evaluated using FerroOrange (Cell Signaling Technology, Danvers, MA, USA; Cat. No. 36104S) fluorescent probe. Cells were seeded at 1x10^5^ density in a black plate with glass bottom. After removing the supernatant media, the cells were washed three times with serum-free medium. A working solution of 1 μmol/L FerroOrange fluorescent probe, prepared in serum-free medium, was then added to the cells. The cells were incubated at 37 °C in dark for 30 min. The cells were observed, and images were acquired using a fluorescence microscope at excitation/emission maxima of 543/580 nm. The fluorescence intensity of FerroOrange was quantified using ImageJ software/9 (National Institutes of Health, Bethesda, MD, USA).

### 2.13 Statistical analyses

Survival outcome differences were plotted using the Kaplan-Meier method and hazard ratios were estimated using Cox proportional hazards modeling. Overall survival (OS) was defined as the length of time (in months) from the date of surgery to the date of death from any cause. One-and two-way Anova followed by Tukey post hoc test was performed to assess the mean differences between groups for one or multiple variables. For the *in vitro* studies, changes in tumor cell growth were analyzed using linear regression model. All the statistical analysis was carried out using GraphPad Prism V.10.6.1 for Windows (GraphPad Software, San Diego, California, USA) and statistical significance was defined as *p* < 0.05.

## 3 Results

**Fig. 1:**
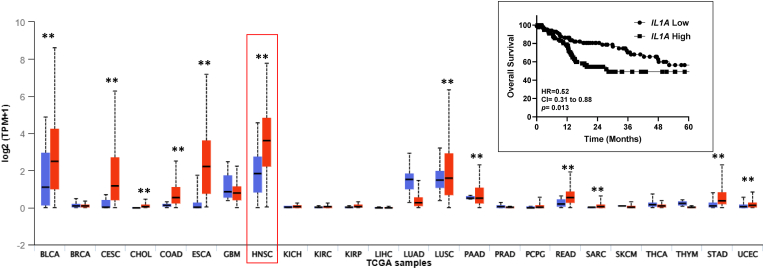
**Expression of *IL1A* in HNSCC.***IL1A* mRNA gene expression among different cancers (including associated normal tissue) was displayed using the UALCAN database. Inset shows Kaplan-Meier estimates of Overall Survival according to high *IL1A* expression (n = 140) and low *IL1A* expression (n = 142) HNSCC patients. HR, hazard ratio; CI, 95% confidence interval, ∗∗p < 0.05.

**Increased *IL1A* is associated with poor survival in HNSCC patients.** Analysis of TCGA mRNA gene expression data using the UALCAN database showed that when compared with corresponding normal tissue, higher *IL1A* transcript levels were observed in the majority of cancer types, including bladder urothelial carcinoma (BLCA); cervical squamous cell carcinomas (CESC), cholangiocarcinoma (CHOL); colon adenocarcinoma (COAD); esophageal carcinoma (ESCA), HNSCC and others using pan-cancer comparison (Fig. 1). Of these cancers, HNSCCs ranked the highest in *IL1A* expression (Fig. 1) and survival rates of high *IL1A* expression patients (median survival = 28.3 months) were significantly lower than those of the low *IL1A* expression patients (median survival = 68.5 months, p = 0.013 Fig. 1 inset). These data support previous data proposing *IL1A* as a prognostic factor in HNSCC.

**Fig. 2:**
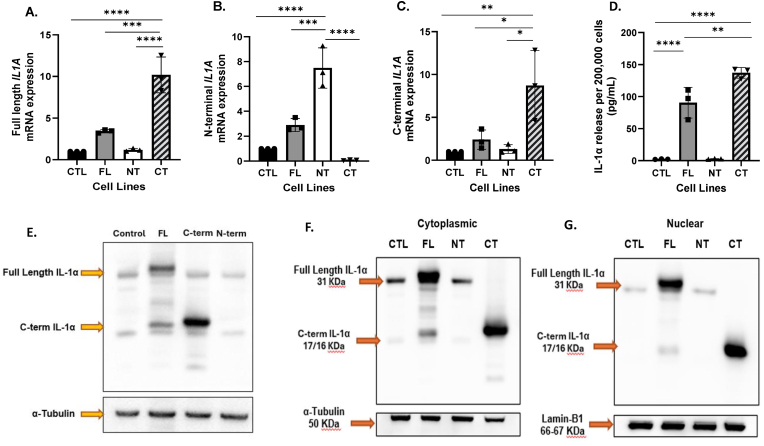
**Characterization of IL-1α overexpression constructs in Cal27 HNSCC cells.** Gene expression (A-C), protein release (D) and protein expression (E-G) of the three IL1A constructs – Full- Length (FL), N-terminal (NT) and C-terminal (CT) in the Cal27 IL-1α-overexpressing cells were analyzed by RT-qPCR, ELISA and Western blot respectively compared to control. GAPDH was used as an endogenous control for PCR analysis (A-C); α-Tubulin was used as the loading control for whole cell lysate and cytoplasmic fraction western blots (E-F); and Lamin B1 was used as the loading control for the nuclear fraction Western blot (G). Bars represent mean ± SEM from n = 3 independent experiments. IL-1α band intensities were normalized to the respective loading controls. ∗p < 0.05; ∗∗p < 0.01; ∗∗∗p < 0.001; ∗∗∗∗p < 0.0001.

**Characterization of IL-1α overexpression constructs in Cal27 HNSCC cells.** Validation of *IL1A* overexpression in the Cal27 cells (FL, NT, CT) by PCR showed that full-length *IL1A* was increased 3.4 fold in FL cells (although not statistically significant (p = 0.09) (Fig. 2A); N-terminal *IL1A* was significantly increased 7.5 fold (p < 0.0001) in NT cells (Fig. 2B); and C-terminal *IL1A* was significantly increased 8.7 (p = 0.0097) fold in CT cells (Fig. 2C) compared to control (CTL). CT cells also showed a significant increase in full-length *IL1A* compared to both the CTL (p < 0.0001) and FL cells (p = 0.0003) (Fig. 2A) and a dramatic (but not significant, p = 0.57) decrease in N-terminal *IL1A* compared to control (Fig. 2B). ELISA results indicated that the FL and CT cells significantly increased IL-1α release into the cell culture media compared to CTL (mean FL: 90.3 pg/mL vs mean CTL: 2.4 pg/mL, p < 0.0001; mean CT: 137 pg/mL vs mean CTL 2.4 pg/mL, p < 0.0001), with CT cells releasing significantly more than FL cells (p = 0.0084) (Fig. 2D). NT cells did not show an increase in IL-1α compared to control (Fig. 2D). Protein expression in whole cell lysates by Western blot showed that expression of full-length IL-1α was increased in FL cells compared to control (Fig. 2E) and that this increased expression was maintained in both the cytoplasmic (Fig. 2F) and nuclear fractions (Fig. 2G). Increased expression of N-terminal IL-1α in NT cells was not observed in the nuclear fraction compared to control (Fig. 2G); and overall IL-1α expression in NT cells appeared to be generally decreased compared to control (Fig. 2E). C-terminal IL-1α expression in the CT cells increased in the whole cell lysates and cytoplasmic fractions compared to control (Fig. 2E and F). C-terminal IL-1α was also observed in the nuclear fraction despite not possessing a NLS (Fig. 2G). These results suggest that the 3 cell lines (FL, CT, NT) were successfully transduced with their respective *IL1A* gene constructs by PCR, although overexpression of IL-1α protein was only demonstrated in FL and CT cell lines.

**Fig. 3:**
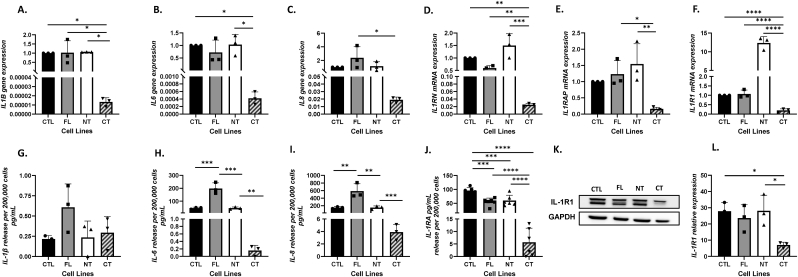
**IL-1α overexpression alters expression of IL-1 pathway genes.** Gene expression of IL1B (A), IL6 (B), IL8 (C), IL1RN (D), IL1RAP (E) and IL1R1 (F) in the three IL1A constructs – Full-Length (FL), N-terminal (NT) and C-terminal (CT) in Cal27 IL-1αoverexpressing cells were analyzed by RT-PCR using GAPDH as an endogenous control. Protein secretion of IL-1β (G), IL-6 (H), IL-8 (I), and IL1RA (J) in cell culture supernatants was quantified by ELISA with protein concentrations normalized to cell numbers. Whole cell lysates were analyzed for IL-1R1 expression by Western blot using GAPDH as a loading control (K). Bars represent mean ± SEM from n = 3 independent experiments.∗p < 0.05; ∗∗p < 0.01; ∗∗∗p < 0.001; ∗∗∗∗p < 0.0001.

**IL-1α overexpression alters expression of IL-1 pathway genes**. Given that FL and CT cells showed increased IL-1α protein expression (Fig. 2D–G), we further looked at the expression of IL-1α family genes and downstream endpoints. There was no increase in gene expression of *IL1B*, *IL6, IL8, IL1R1, IL1RN*, and *IL1RAP* in FL compared to CTL (Fig. 3A–F). However, there was a significant increase in protein release of IL-6 (mean FL: 198.1 pg/mL vs mean CTL: 46.4 pg/mL, p = 0.0002, Fig. 3H), and IL-8 (mean FL: 584.6 pg/mL vs mean CTL: 150.4 pg/mL, p = 0.0033, Fig. 3I) suggesting that the IL-1α released from FL cells was active and led to activation of downstream signaling. To confirm biological activity, we further showed that the increase in IL-1α from FL cells could be suppressed by anakinra (human recombinant IL-1RA) (Supplemental Fig. 1A) and neutralizing antibodies to IL-1α but not IL-1β (Supplemental Fig. 1B). There was also a significant decrease in release of IL-1RA in FL compared to CTL (mean FL: 55.1 pg/mL vs mean CTL: 95.6 pg/mL, p = 0.0002, Fig. 3J). There were no significant increases in NT cells in any of the parameters measured with exception of the IL-1R1 gene expression (12.3 fold, p < 0.0001, Fig. 3F) compared to CTL. Additionally, there was no change in IL-1β protein secretion among the cell lines (Fig. 3G). CT cells showed a significant decrease in *IL1B, IL6, IL1R1, and IL1RN* gene expression compared to CTL (p < 0.05), a significant decrease in *IL1B, IL8, IL1R1, IL1RAP, and IL1RN* gene expression compared to FL (p < 0.05), and a significant decrease in *IL1B, IL6, IL1R1, IL1RAP, and IL1RN* gene expression compared to NT cells (p < 0.05) (Fig. 3A–F). This general trend also remained with decreased protein release in CT cells of IL-6, IL-8 and IL-1RA (Fig. 3H–J). To understand why CT cells lacked an increase in IL-1 pathway endpoints (*i.e*. IL-6 and IL-8, Fig. 3B,C,H,I), we analyzed differences in IL-1R1 protein expression and showed a significant decrease in IL-1R1 expression in CT cells compared to all of the other lines (p < 0.05, Fig. 3K and L). These results indicate that overexpression of full-length and C-terminal IL-1α leads to completely different outcomes in IL-1 pathway signaling.

**Fig. 4:**
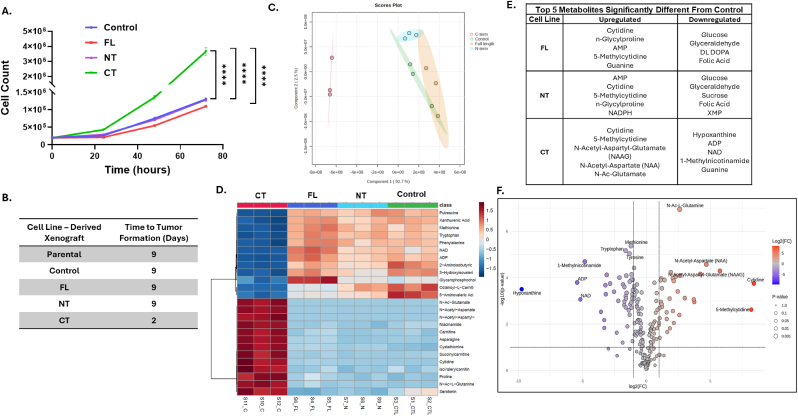
**C-terminal IL-1α overexpressing cells demonstrate metabolic changes.** Changes in proliferation of the control and three IL-1αoverexpressing cell lines (Full-Length (FL), N-terminal (NT), and C-terminal (CT)) were determined by counting cells over time (A). Time to xenograft tumor formation (3 mm) after cell line inoculation in nude mice was recorded (B). Metabolomics was conducted on the control and three IL-1αoverexpressing cell lines (C–F). Partial least squares discriminant analysis (PLS-DA) (C) and heat map show metabolic separation and unique differences in metabolite profiles (D) among cell lines. E: Shown are the top five upregulated and downregulated metabolites compared to control cells (E). Metabolites significantly upregulated and downregulated in C-term IL-1α overexpressing cells compared to control cells are shown by volcano plot (F). Bars represent mean ± SEM from n = 3 independent experiments. ∗p < 0.05; ∗∗p < 0.01; ∗∗∗p < 0.001; ∗∗∗∗p < 0.0001.

**C-terminal IL-1α overexpressing cells demonstrate metabolic changes.** To determine the biological consequences of IL-1α overexpression, we first compared the growth rate of the CT cells and the 3 IL-1α-overexpressing cell lines (FL, N, CT) *in vitro*. We observed that the growth rate of the CT cells was significantly faster than the other cell lines (p < 0.0001) and there was no difference in growth rates among the CTL, FL and NT cell lines (Fig. 4A). When these 4 cell lines were inoculated into the flanks of nude mice, CT cells began to form palpable tumors on Day 2 compared to Day 9 from the other inoculated cell lines (Fig. 4B) suggesting that the CT cells were more aggressive than the other cell lines. Metabolomic analysis of the 4 cell lines revealed a clear separation of the CT cells in global metabolite expression and metabolite signature compared to the other cell lines according to PLS-DA analysis (Fig. 4C) and the constructed heatmap (Fig. 4D). In all the IL-1α overexpressing cells (FL, NT and CT), cytidine and 5-methyl cytidine were significantly upregulated compared to CTL cells (p < 0.05, Fig. 4E). However, CT cells additionally upregulated N-Acetyl-Aspartyl-Glutamate (NAAG), N-Acetyl-Aspartate (NAA) and N-Ac-Glutamate (NAG) suggesting a dependency on glutamate or glutamine metabolism (Fig. 4E and F) that is unique to CT IL-1α-overexpressing cells. Similar to the metabolomic analysis, transcriptomic analyses using single cell RNA sequencing revealed a clear separation of the CT cells compared to the other cell lines in the PCA analysis (Supplementary Fig. 2A). KEGG pathway enrichment from CT vs control cells showed upregulated genes from the MAPK, Epstein Barr virus infection and lipid/atherosclerosis pathways (Supplementary Fig. 2B); and downregulated genes from the ribonucleoprotein complex biogenesis, chromosomal region, and centrosome pathways (Supplementary Fig. 2C). Gene ontology (GO) pathway enrichment from CT versus control cells showed upregulated genes from the cell cycle, Wnt, and axon guidance pathways (Supplementary Fig. 2D); and downregulated genes from cell adhesion and neutrophil activation-related pathways (Supplementary Fig. 2E). Top 20 upregulated and downregulated genes from CT versus control cells are listed in Supplementary Table 3. Together these results suggest that overexpression of the C-terminal of IL-1α leads to genetic and metabolic changes that result in an aggressive phenotype *in vitro* and *in vivo*.

**Fig. 5:**
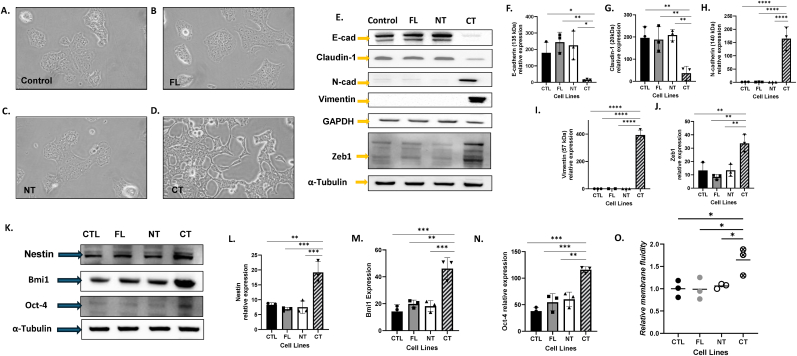
**IL-1α overexpression induces changes in cell morphology, EMT and stemness markers.** Morphological changes were imaged by phase-contrast microscopy showing control (A) and full-length (FL) (B), N-terminal (NT) (C) and C-terminal (CT) (D) IL-1α overexpressed cells. Whole cell lysates are analyzed for E-cadherin (E-cad), Claudin-1, N-cadherin (N-cad), Vimentin and ZEB-1 expression by Western blot using GAPDH and α-Tubulin as a loading control (E) and quantification of the protein expression is shown in F-J. Whole cell lysates were analyzed for stemness markers (Nestin, BMI-1 and OCT-4) by Western blot using α-Tubulin as a loading control (K) and quantification of the protein expression is shown in L-N. Changes in membrane fluidity was measured using a fluorescent lipophilic pyrene probe before flow cytometry analysis (O). Bars represent mean ± SEM from n = 3 independent experiments. ∗p < 0.05; ∗∗p < 0.01; ∗∗∗p < 0.001; ∗∗∗∗p < 0.0001.

**IL-1α overexpression induces changes in cell morphology, EMT and stemness markers.** As vimentin (VIM), which is important in the epithelial to mesenchymal transition (EMT) process [41,42], was one of the top genes upregulated in the CT cells compared to control **(**Supplementary Table 3**)**, we next investigated any changes in cell morphology and EMT markers. Compared to the FL, NT and control cells, CT cells displayed an elongated morphology with spindle like protrusions characteristic of mesenchymal cells (Fig. 5A–D). Consistent with the transcriptomic findings, the mesenchymal marker VIM was prominently expressed in CT cells, in addition to the other mesenchymal markers N-cadherin and Zeb1 which was nearly undetectable in the other cell lines (p < 0.05, Fig. 5E–G, I,J). Epithelial markers such as E-cadherin and claudin were present in the FL, NT and CTL cells but absent in the CT cells (Fig. 5E, F, G) suggesting that the CT cells had undergone EMT. Given that EMT can induce stemness properties, we next investigated the differential expression of stem cell markers (nestin, Bmi1 and oct-4) in the 4 cell lines. All three markers were significantly increased in the CT cells compared to the other cell lines (p < 0.01, Fig. 5K–N). Lastly since EMT is associated with changes in lipid content during development [43]), we analyzed membrane fluidity as a potential biophysical divergence in the CT cells. This was done by monitoring the incorporation of a fluorescent lipophilic pyrene probe into the cell membrane of each of the 4 cell lines. We found that the membrane fluidity of CT cells was significantly higher than the other cell lines (p < 0.05, Fig. 5O). These results suggest that overexpression of the C-terminal of IL-1α leads to EMT and stem-like properties that may drive the aggressive phenotype of these cells.

**Fig. 6:**
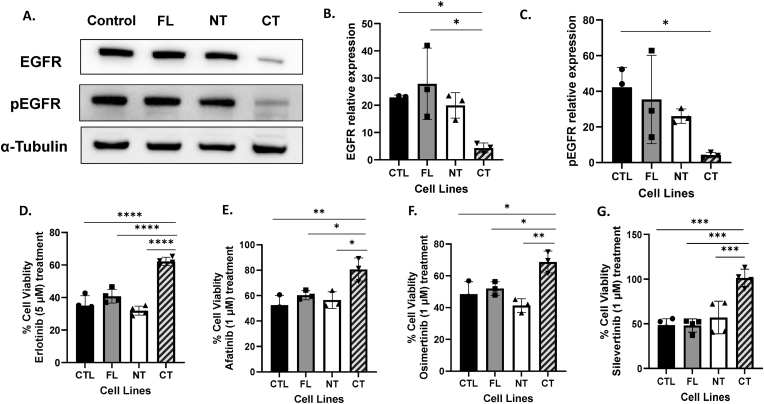
**IL-1α overexpression induces resistance to EGFR tyrosine kinase inhibitors.** (A) Whole cell lysates were analyzed for EGFR and phosphorylated EGFR (pEGFR) expression by Western blot in control (CTL) and full-length (FL), N-terminal (NT) and C-terminal (CT) IL-1α overexpressed cells using α-Tubulin as a loading control, with quantification of protein expression shown in B and C. (D-G) Cell viability following 48 h treatment with Erlotinib (5 μM) (D), Afatinib (1 μM) (E), Osimertinib (1 μM) (F) and Silevertinib (1 μM) (G) was measured using MTT assays. Bars represent mean ± SEM from n = 3 independent experiments. ∗p < 0.05; ∗∗p < 0.01; ∗∗∗p < 0.001; ∗∗∗∗p < 0.0001.

**IL-1α overexpression suppresses EGFR expression leading to resistance to EGFR tyrosine kinase inhibitors (TKIs).** Prior studies in NSCLC have shown that EMT can induce resistance to EGFR-TKIs [44]. We therefore analyzed if the EMT-like properties observed in the CT cells (Fig. 5) conferred resistance to the EGFR TKIs – erlotinib, afatinib, osimertinib and silevertinib. EGFR and phospho-EGFR expression was dramatically decreased in the CT cells (p < 0.05, Fig. 6A–C) but not in the FL and NT cells compared to CTL. We also found that for all the analyzed EGFR TKIs, there was significantly less cell killing in the CT cells compared to the other cell lines (CTL, FL, NT) (p < 0.05, Fig. 6D–G, Supplemental Fig. 3) suggesting that the EMT induced by C-terminal IL-1α overexpression may contribute to resistance to EGFR TKIs.

**Fig. 7:**
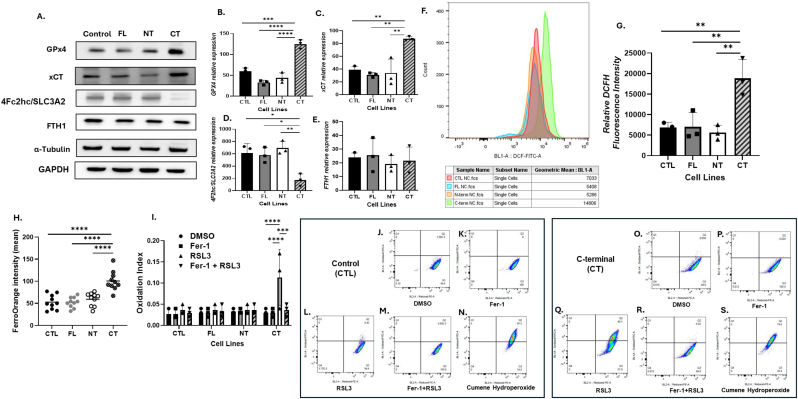
**IL-1α overexpress sion induces a ferroptotic environment.** (A) Whole cell lysates were analyzed for GPX4, xCT, 4F2hc/SLC3A2 and FTH1 expression in the control (CTL) and full-length (FL), N-terminal (NT) and C-terminal (CT) IL-1α overexpressed cells by Western blot using α-tubulin and GAPDH as loading controls, and quantification of the associated protein expression is shown in B-E. (F) Total reactive oxygen species in the cell lines were measured by DCFH staining. (G) Intracellular Fe2+ levels were measured by changes in FerroOrange fluorescence. (H) Lipid peroxidation was assessed using BODIPY C11 fluorescent probe, shown as oxidation index following 24 h treatment with Vehicle (DMSO), Fer-1 (1 μM), RSL3 (1 μM) and Fer-1 (1 μM) + RSL3 (1 μM). Representative quadrant dot plots of BODIPY C11 fluorescence are shown for control (CTL) cells are shown in I-M; and for C-terminal (CT) cells in N–R. Bars represent mean ± SEM from n = 3 independent experiments. ∗p < 0.05; ∗∗p < 0.01; ∗∗∗p < 0.001; ∗∗∗∗p < 0.0001.

**IL-1α overexpression induces ferroptosis-associated remodeling.** In previous reports, the mesenchymal marker Zeb1 observed in our CT cells (Fig. 5E) has been associated with vulnerability to ferroptosis [45]. We therefore investigated differences in markers associated with a ferroptotic environment in CTL and the three IL-1α-overexpressing cell lines. Our results showed that glutathione peroxidase 4 (GPx4) (Fig. 7A and B) and xCT (Fig. 7A–C) expression were significantly increased (p < 0.01) in the CT cell lines compared to the other cell lines. xCT, which is the light chain subunit of the xCT transporter, cooperates with the heavy chain subunit (4Fc2hc/SLC3A2) to form the functional xCT transporter [46]. However, expression of 4Fc2hc/SLC3A2 was significantly decreased in CT cells (p < 0.01, Fig. 7A–D) compared to the other cell lines suggesting a potential impairment of cystine/glutamate exchange. There was no change in Ferritin Heavy Chain 1 (FTH1) expression among the cell lines (Fig. 7A–E). Intracellular reactive oxygen species production indicated that the CT cells have increased steady-state levels of intracellular oxidants compared to the other cell lines (p < 0.01, Fig. 7F and G). Congruently, intracellular iron (p < 0.0001, Fig. 7H) and intracellular lipid peroxidation (oxidized vs reduced state) induced by RSL3 (GPx4 inhibitor) using significantly increased (p < 0.0001) in CT cells relative to all other cell lines, which was completely suppressed by Fer-1 (lipid peroxidation inhibitor) (p < 0.001, Fig. 7I–S). Together these results suggest a highly oxidative environment that is associated with increased redox-active iron and lipid peroxidation in CT cells, which is characteristic of a ferroptotic environment.

**Fig. 8:**
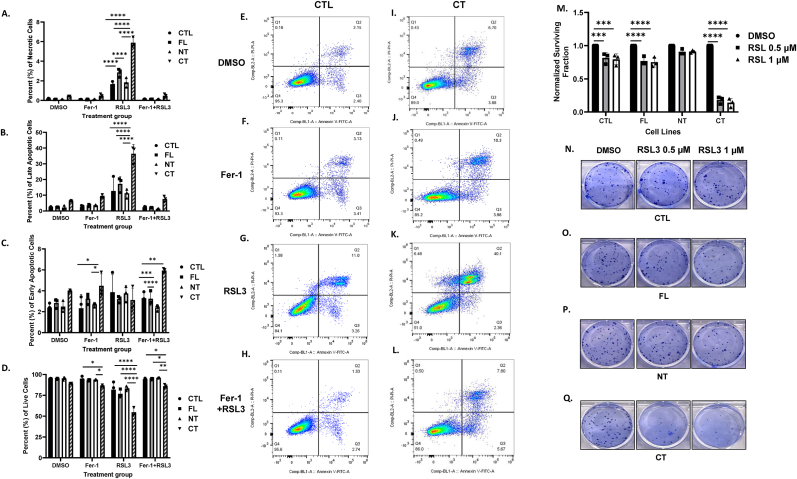
**IL-1α overexpression triggers sensitivity to RSL3.** (A-L) Cell response to RSL3 with and without ferrostatin (Fer-1) was assessed using Annexin V-PI staining. Quantification of necrotic (A), late apoptotic (B), early apoptotic (C), and live (D) cell populations is shown following 24 h treatment with vehicle (DMSO), Fer-1 (1 μM), RSL3 (1 μM), or Fer-1 (1 μM) + RSL3 (1 μM) in control (CTL) and full-length (FL), N-terminal (NT) and C-terminal (CT) IL-1α overexpressing cells. Representative Annexin V-PI quadrant dot plots are shown for vehicles and treated CTL (E-H) and CT cells (I-L). Clonogenic survival was assessed in the CTL and IL-1α overexpressing cells treated with vehicle (DMSO), 0.5 μM RSL3, and 1 μM RSL3. The surviving fractions are shown in bar graph (M) and representative colony formation images are shown for CTL (N), FL (O), NT (P), and CT (Q) IL-1α overexpressing cells. Bars represent mean ± SEM from n = 3 independent experiments. ∗p < 0.05; ∗∗p < 0.01; ∗∗∗p < 0.001; ∗∗∗∗p < 0.0001.

**IL-1α overexpression increases sensitivity to RSL3-mediated ferroptosis.** Given the increased ferroptotic environment in CT cells (Fig. 7) we next examined the sensitivity of the CT cells to RSL3 compared to the other cell lines. Using the Annexin V/PI assay we observed a significant increase in necrotic (p < 0.0001, Fig. 8A–C, E), and late apoptotic cells (p < 0.0001, Fig. 8A–C, F); and a significant decrease in live cells (p < 0.0001, Fig. 8A–C, H) following RSL3 treatment in the CT cells compared to CTL and the other cell lines. The FL cells also showed a significant increase in necrotic cells compared to CTL (p < 0.0001) but was not as dramatic as the CT cells (Fig. 8E). The addition of ferrostatin (Fig. 8B) completely reversed the effect of RSL3 in the CT cells confirming RSL3-mediated ferroptosis induction in the CT cells (Fig. 8D–H). Clonogenic analysis additionally revealed the increased sensitivity of the CT cells to RSL3 compared to the other cell lines (p < 0.0001, Fig. 8I–M). Altogether, it appears that increased CT expression conferred an increased vulnerability to ferroptosis induction.

## 4 Discussion

Overall, the presented results suggest that the association of increased tumor-associated IL-1α expression with poor survival outcomes may be due to the development of a ferroptotic environment (Fig. 7) and EMT (Fig. 5), leading to more aggressive (Fig. 4), therapy-resistant disease (Fig. 6). Of note, the ferrototic environment was only observed in cells overexpressing the C-terminal (CT) of IL-1α (and not the full-length IL-1α) (Fig. 7) even though both CT and FL overexpressing cells secreted IL-1α, as measured by ELISA (Fig. 2D) likely because the C-terminal of IL-1α is more active than full-length IL-1α. For example, although both forms of IL-1α (FL and CT) are able to bind to the IL-1R1 and trigger downstream signaling, the CT form is a high-affinity IL-1R1 agonist, and binds to the IL-1R1 several folds more strongly than the FL form [47,48]. FL IL-1α tends to preferentially function intracellularly particularly in the nucleus in an IL-1R1-independent manner, where it can produce transcriptional changes, and induce survival programs including IL-6 and IL-8 [48,49]. Therefore, sustained expression and secretion of the more bioactive CT form may have triggered the development of the intracellular ferroptotic environment.

In the CT cells, evidence suggested that the observed ferroptotic environment was due to the increase in membrane fluidity accompanied by the loss of expression of the membranous receptors IL-1R1 (Fig. 3K) and EGFR (Fig. 6A–C) compared to the other cells. Ferroptosis is a form of non-apoptotic cell death driven by excessive iron-catalyzed oxidation of polyunsaturated fatty acid-containing phospholipids [50]. Following up on this evidence, we observed that the CT cells did show increased levels of intracellular iron (Fig. 7H), steady-state oxidation (Fig. 7G) and lipid peroxidation (Fig. 7I–S) supporting a ferroptotic environment. Excessive lipid peroxidation in cellular membranes changes the physical properties of lipids including thinning the membrane [51], increasing local curvature [52] and loss of anchoring of membranous proteins [53] making membranes more prone to rupturing and cell death. Although ferroptosis is known to result in cell death, it has recently begun to emerge that tumor cells that can - survive ferroptotic pressure or proliferate under sub-lethal ferroptotic stress can be more aggressive. From a biophysical standpoint, it has recently been reported in glioma cells that with accelerated iron accumulation, cells become significantly less stiff, have greater membrane fluidity, and are significantly more motile [54,55]. Ferroptosis evasion can be characterized by the following features: 1 - an increased dependency on GPx4 activity, which is necessary for the neutralization of lipid hydroperoxides; 2 – glutathione (GSH) synthesis, which is the cofactor for GPx4 activity; 3. sustained iron retention, which can catalyze lipid peroxidation to facilitate biophysical remodeling; and 4 - endogenous glutamate, which is necessary to form GSH (in addition to glycine and cysteine) [56,57]. Indeed, we observed elevated iron levels, lipid peroxidation, and increased GPX4 expression to counteract the increase lipid peroxidation (Fig. 7A) in the CT cells further supporting an adaptive survival mechanism to facilitate ferroptosis evasion. Therefore, it appears that IL-1α overexpression causes a robust cellular remodeling that allows tumor cells to leverage ferroptosis to become more aggressive and potentially more difficult to treat which can lead to poor therapy response and survival outcomes.

Ferroptotic pressure is also associated with EMT which drives transcription factors including ZEB1 [45] which was dramatically increased in the CT cells (Fig. 5E). Stabilization of these transcription factors lead to a decrease in epithelial genes (e.g. E-cadherin) involved in the loss of cellular adhesion, and an increase in mesenchymal genes (e.g. N-cadherin, vimentin) involved in the gain of migratory properties - crucial for cancer metastasis [58]. Again, these features were prominent in the CT cells (Fig. 5E). In fact, vimentin gene expression was the 4th most upregulated gene in the CT cells compared to control cells (Supplemental Table 3) which was not present in any of the other cell lines. EMT has been shown to trigger cystine uptake, GSH synthesis and lipid remodeling allowing cells to survive ferroptotic pressure [59]. Therefore, it is likely that EMT observed in the CT cells enabled them to survive and proliferate in ferroptotic conditions.

One interesting finding is the presence of increased NAAG, NAA and NAG from the metabolomics analyses in the CT cells compared to the other IL-1α overexpressed cells (Fig. 4E and F). NAAG metabolism is important particularly in the mammalian nervous system where it acts as a neurotransmitter in the brain [60]. NAAG can be broken down into NAA and glutamate by glutamate carboxypeptidase II (GCPII), and the rate of this reaction is critical for controlling synaptic glutamate levels [60]. Given the necessity of glutamate for GSH synthesis and ultimately GPX4 activity, it is possible that the CT cells are using alternate sources (*i.e.* NAAG metabolism) in addition to glutaminolysis to obtain glutamate to combat the ferroptotic pressure. Previous results have reported that increased synthesis of NAAG acts as a glutamate reservoir in high grade ovarian and brain cancers for when glutamate production from other sources is limited [61,62]. Additionally, Asaka et al. (2019) showed that the inhibition of GCPII suppresses tumor growth in patient-derived recurrent ovarian cancer orthotopic tumors and reduces glutamate production [63]. These data support our current findings that NAAG metabolism may act as a source of glutamate in the CT cells to persist despite the ferroptotic environment.

The observed increase in the metabolite NAG in the CT cells (Fig. 4E and F) is somewhat unclear. NAG is an essential allosteric activator of carbamylphosphate synthetase (CPS1), the rate-limiting enzyme of the urea cycle [64]. Mitochondrial N-acetylglutamate synthetase (NAGS) catalyzes the reaction of glutamate and acetyl-CoA to form NAG and CoA [65]. NAGS is also activated by arginine [66]. Previous studies have proposed that the urea cycle driven by arginine can promote resistance to ferroptosis in non-small cell lung cancer (NSCLC) cells [67] by blocking cytosolic glutamine from entering the oxidative tricarboxylic acid (TCA) cycle and reducing mitochondrial-derived lipid ROS [67,68]. Therefore, increased NAG may be another mechanism (in addition to increased NAAG metabolism) that CT cells use to combat the oxidative ferroptotic environment.

Although we found that increased IL-1α expression in the CT cells was associated with lipid peroxidation, it is not clear how IL-1α is metabolically inducing lipid peroxidation. The most obvious source is the elevated iron levels in the CT cells, as iron-catalyzed lipid peroxidation is the foundation of ferroptosis induction. This is further corroborated by the enhanced sensitivity to ferroptosis induction. However, the mechanism by which IL-1α is elevating iron levels is unclear. Given that IL-1α is known to induce nitric oxide synthase (iNOS/NOS2) and nitric oxide (NO) production can promote iron accumulation due to its ability to bind iron and disrupt aconitase activity [69,70], this may be a logical cause. In support of this, we observed a significant increase in NOS2 gene expression in the CT cells compared to other overexpressed and control cells and in the RNA sequencing data when comparing CT vs control cells (Supplementary Fig. 7). It is also possible that IL-1α is increasing the production of peroxinitrite [71], which is a highly reactive nitrogen species formed from the reaction of NO with superoxide that can catalyze lipid peroxidation [72]. However, this introduces the complexity of iron/nitric oxide/peroxynitrite chemistry as a confounding variable as iron can readily react with nitric oxide (∼10^7^ – 10^8^ M^−1^ s^−1^) to prevent peroxynitrite formation. Iron can also react with and reduce peroxynitrite directly (∼10^6^ – 10^7^ M^−1^ s^−1^), however, its breakdown products (HO^•^ and NO_2_^•^) can readily initiate lipid peroxidation if formed within the vicinity of unsaturated fatty acids (∼10^10^ M^−1^ s^−1^ and 10^5^ M^−1^ s^−1^, respectively) [73,74]. Taken together, it appears that there may be a complex underlying relationship between nitric oxide signaling and iron metabolism that is modulated by IL-1α. Future studies are needed to confirm if IL-1α-induced iNOS activity is involved in the ferroptosis vulnerability observed in the CT cells.

Our findings raise important implications about the utility of IL-1α expression as a predictive biomarker for HNSCC. Previous studies (including ours) in various disease sites have shown that high IL-1α gene and protein expression is correlated with poor survival outcomes especially with increased likelihood of recurrence and metastasis [16] [30,31] [32]. However, IL-1 blockade (anakinra, neutralizing antibodies) as a treatment strategy for cancer has not been successful [75,76] (NCT00072111, NCT03631199, NCT03626545, NCT02492750, NCT01767857). A likely explanation for these failures is the inability of these agents to target intracellular IL-1α. Additionally, the absence of membranous IL-1R1 in the CT cells (Fig. 3K and L) would explain why anakinra may be ineffective on high IL-1α expressing tumor cells. The loss of membranous EGFR expression in the CT cells also suggests that high IL-1α expression would drive resistance to EGFR inhibitors which was demonstrated in response to four generations of EGFR TKIs (Fig. 6D–G). However, the ferroptotic environment existing in the CT cells suggest that they would be highly sensitive to any agent that further drives ferroptosis. In support of this we showed that the CT cells (and not the other cell lines) were highly sensitive to the GPX4 inhibitor - RSL3 (Fig. 8) suggesting that increased IL-1α expression may be correlated with favorable response to GPX4 inhibitors. While theoretically feasible, the development of GPX4 inhibitors for clinical use has been challenged by poor drug bioavailability and poor selectivity (targets other GPX enzymes) [77,78]. Given our findings, targeting other major players in the ferroptotic process i.e. Xct transporter, NAAG metabolism, NAG or arginine may be potential targets for further study in high IL-1α expressing tumor cells.

Although not addressed in these studies, high IL-1α expression may be associated with an immunosuppressive tumor microenvironment *in vivo*. One of the limitations of these studies is that we did not collect and analyze immunosuppressive and stromal differences in the tumor microenvironment of the IL-1α-overexpressing tumor xenografts after the termination of the study (Fig. 4B). IL-1α is known to drive the expansion of myeloid-derived suppressor cells (MDSCs), skews tumor-associated macrophages (TAMs) toward an M2-like phenotype, increases immune checkpoint (e.g. PD-L1) expression, activates cancer-associated fibroblasts and drives exclusion of cytotoxic CD8^+^ T cells [79,80]. This suggests that strategies that can reverse the immunosuppressive environment in addition to driving ferroptosis would be helpful for cancer therapy.

Altogether our results provide a mechanistic argument for why increased tumor-associated IL-1α expression may be associated with poor survival outcomes with an emphasis on iron-mediated lipid peroxidation and EMT. Therefore, high IL-1α-expressing tumors may be susceptible to agents that further increase lipid peroxidation. Although more investigations should be carried out *in vitro* and *in vivo* in this area to confirm these ideas; but if consistent, this work could highlight tumor-associated IL-1α expression as a novel predictive marker of drug response in HNSCCs.

## Ethics approval and consent to participate

All the experimental methods were conducted in compliance with relevant guidelines and regulations. Animal experiments were approved by the Institutional Animal Care and Use Committee (IACUC), University of Iowa (Protocol number 3102564-001). No human subjects were involved in this study.

## Funding

This work was funded by Merit Review Award #2I01BX004829 from the United States (U.S.) Department of Veterans Affairs Biomedical Laboratory Research and Development Service.

## CRediT authorship contribution statement

**Ishrat Nourin Khan:** Conceptualization, Data curation, Formal analysis, Investigation, Methodology, Validation, Visualization, Writing – original draft, Writing – review & editing. **Krishna Awasthi:** Data curation, Formal analysis, Methodology, Validation, Writing – review & editing. **Ziyu Wang:** Data curation, Formal analysis, Methodology, Validation, Writing – review & editing. **Nafis Md Irfan:** Data curation, Formal analysis, Methodology, Validation, Visualization, Writing – review & editing. **Jay Saepoo:** Data curation, Formal analysis, Methodology, Validation, Visualization, Writing – review & editing. **Joan N. Whittier:** Data curation, Formal analysis, Methodology, Validation, Visualization, Writing – review & editing. **Nurgul Koyuncu:** Data curation, Formal analysis, Methodology, Validation, Visualization, Writing – review & editing. **Md Roman Mogal:** Data curation, Formal analysis, Methodology, Validation, Visualization, Writing – review & editing. **M.M. Hasibuzzaman:** Conceptualization, Writing – review & editing. **Shujie Yang:** Conceptualization, Investigation, Resources, Writing – review & editing. **Michael Petronek:** Investigation, Methodology, Resources, Validation, Visualization, Writing – review & editing. **Andrean L. Simons:** Conceptualization, Formal analysis, Funding acquisition, Investigation, Methodology, Project administration, Resources, Software, Supervision, Writing – original draft, Writing – review & editing.

## Declaration of competing interest

The authors declare no competing interests.

## Data Availability

Data will be made available on request.
