# Supplementary material for: C-terminal interleukin 1 alpha (IL-1α) overexpression drives EMT and a vulnerability to ferroptosis in HNSCC
Source: Redox Biol. 2026 Apr 16;93:104172. doi: 10.1016/j.redox.2026.104172 (PMC13122707; doi:10.1016/j.redox.2026.104172)
Supplement: Multimedia component 1 [file mmc1.docx]

**Supplementary Figure Legends**

**Supplementary Figure 1: Validation of IL-1α biological activity.** Secretion of IL-6 protein in cell culture supernatant were measured by ELISA after treating IL-1α-overexpressed Cal27 cells with anakinra (0.5, 1 and 5 µg/mL) (A) neutralizing antibodies (1 µg/mL) against IL-1α (nIL-1α ab) and IL-1β (nIL-1β ab) and concentrations were normalized to cell number. Bars represent the mean of *n* = 3 independent experiments. Error bars represent standard error from the mean. ****p*<0.001; *****p*<.0001.

**Supplementary Figure 2: C-terminal IL-1α overexpression induces unique transcriptional changes compared to other IL-1α overexpressing cells.** RNA sequencing was performed on control and three IL-1α overexpressed cell lines. (**A**) Principal Component Analysis (PCA) plot displays the clustering of samples based on transcriptomics differences. Kyoto Encyclopedia of Genes and Genomes (KEGG) and Gene Ontology (GO) enrichment dot plot representing the top 20 upregulated pathways (**B, D**) and the top 20 downregulated pathways (**C,E**) in C-terminal IL-1α overexpressed cell lines compared to control cells. Bars represent mean ± SEM from *n* = 3 independent experiments. **p* <.05; ***p* < .01; ****p* < .001; *****p* < .0001.

**Supplementary Figure 3: IL-1α overexpression induces resistance to EGFR tyrosine kinase inhibitors.** (A-D) Cell viability following 48 h treatment with Erlotinib, Afatinib, Osimertinib and Sileveretinib in three different doses (0.5, 1 and 5 µM) were measured using MTT assays. Bars represent the mean of *n* = 3 independent experiments. Error bars represent standard error from the mean. **p*<0.05, ***p*<0.01,

****p*<0.001; *****p*<.0001.

**Supplementary Figure 4: Related to figure 7: IL-1α overexpression induces a ferroptotic environment.** Total reactive oxygen species in the IL-1α overexpressed cell lines were measured by DCFH staining. (A) Flow cytometry gating strategies of IL-1α overexpressed cells (Control (CTL)) is shown. (B) Total reactive oxygen species as geometric mean is shown in the histogram and (C) relative DCFH fluorescence intensity was shown in the bar graph. Bars represent the mean of *n* = 3 experiments. Error bars represent standard error from the mean. **p* < .05; ***p* < .01; ****p* < .001.

**Supplementary Figure 5: Related to figure 7: IL-1α overexpression induces a ferroptotic environment.** Lipid peroxidation was assessed using BODIPY C11 fluorescent probes. (A) Flow cytometry gating strategies of IL-1α overexpressed cells (Control (CTL)) treated with DMSO is shown. IL-1α overexpressed cells were treated with DMSO (B-E), Fer-1 (F-I), RSL3 (J-M), Fer-1+RSL3 (N-Q) and Cumene hydroperoxide (R-U) and the lipid oxidation is shown in quadrant distribution of dot plot. Bars represent the mean of *n* = 3 experiments. Error bars represent standard error from the mean. **p* < .05; ***p* < .01; ****p* < .00.

**Supplementary Figure 6: Related to figure 8: IL-1α overexpression triggers sensitivity to RSL3.** Annexin V-PI staining was used to assess the IL-1α overexpressed cell’s response to RSL3 with and without ferrostatin (Fer-1). This experiment was assessed using BODIPY C11 fluorescent probes. (A) Flow cytometry gating strategies of IL-1α overexpressed cells (Control (CTL)) treated with DMSO is shown. IL-1α overexpressed cells were treated with DMSO (B,F,J,N), Fer-1 (C,G,K,O), RSL3 (D,H,L,P) and Fer-1+RSL3 (E,I,M,Q) for 24h and quantification of necrotic , late apoptotic, early apoptotic and live cell populations showed in quadrant distribution of dot plot. Bars represent the mean of *n* = 3 experiments. Error bars represent standard error from the mean. **p* < .05; ***p* < .01; ****p* < .001.

**Supplementary Figure 7: iNOS expression of IL-1α overexpression constructs in Cal27 HNSCC cells.** Gene expression of the three IL1A constructs – Full- Length (FL), N-terminal (NT) and C-terminal (CT) in the Cal27 IL-1α-overexpressing cells were analyzed by RT-qPCR, compared to control. GAPDH was used as an endogenous control for PCR analysis (A); (B)RNA-seq analysis showing the Log_2_ Fold change in NOS2 expression in the C-terminal (CT) compared to the Control cells. Bars represent mean ± SEM from n = 3 independent experiments. IL-1α band intensities were normalized to the respective loading controls. *p < .05; **p < .01; ***p < .001; ****p < .0001.
