# Supplementary material for: C-terminal interleukin 1 alpha (IL-1α) overexpression drives EMT and a vulnerability to ferroptosis in HNSCC
Source: Redox Biol. 2026 Apr 16;93:104172. doi: 10.1016/j.redox.2026.104172 (PMC13122707; doi:10.1016/j.redox.2026.104172)
Supplement: Multimedia component 2 [file mmc2.pptx]

## Slide 1
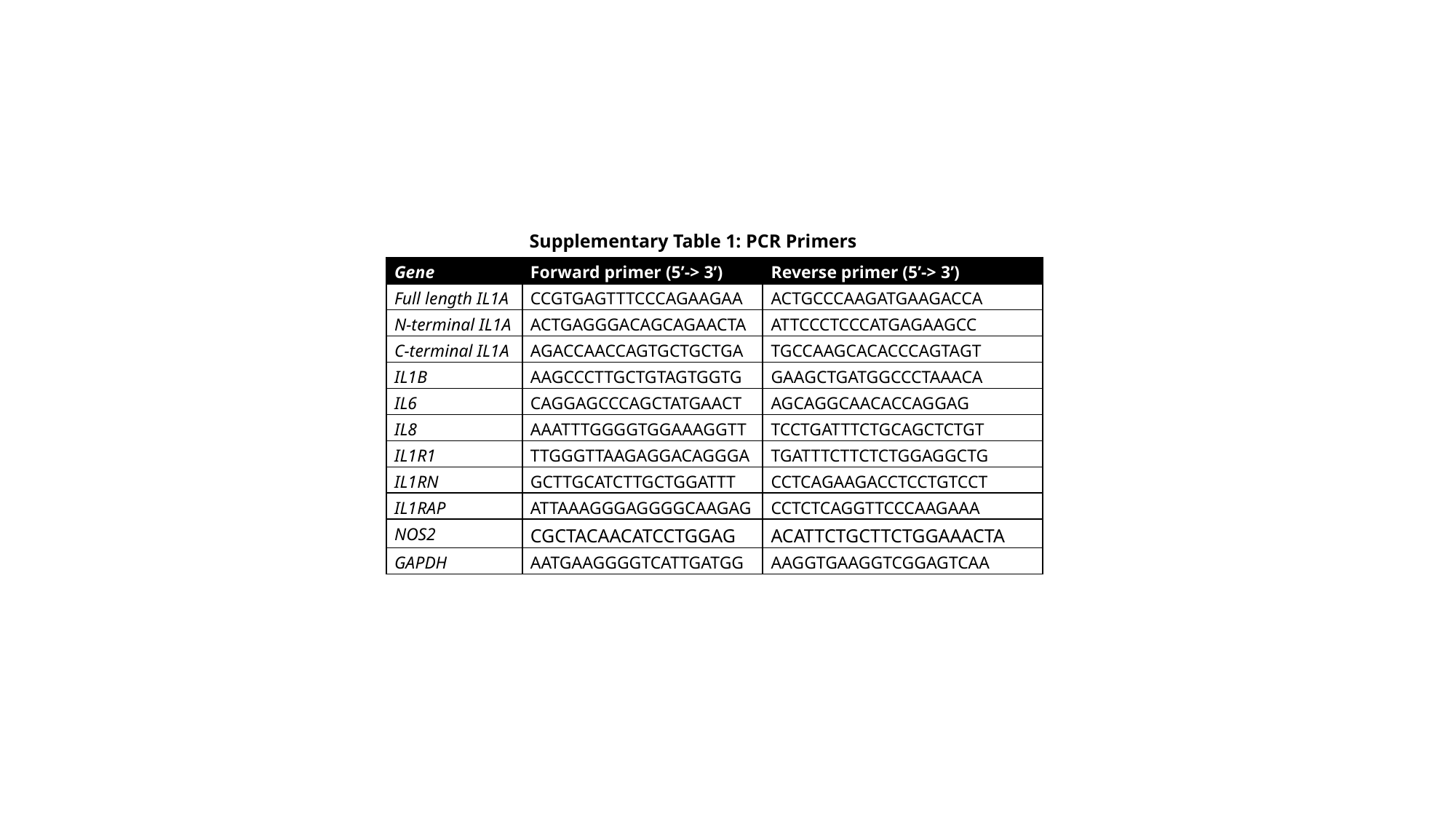

Supplementary Table 1: PCR Primers
| Gene | Forward primer (5’-> 3’) | Reverse primer (5’-> 3’) |
| --- | --- | --- |
| Full length IL1A | CCGTGAGTTTCCCAGAAGAA | ACTGCCCAAGATGAAGACCA |
| N-terminal IL1A | ACTGAGGGACAGCAGAACTA | ATTCCCTCCCATGAGAAGCC |
| C-terminal IL1A | AGACCAACCAGTGCTGCTGA | TGCCAAGCACACCCAGTAGT |
| IL1B | AAGCCCTTGCTGTAGTGGTG | GAAGCTGATGGCCCTAAACA |
| IL6 | CAGGAGCCCAGCTATGAACT | AGCAGGCAACACCAGGAG |
| IL8 | AAATTTGGGGTGGAAAGGTT | TCCTGATTTCTGCAGCTCTGT |
| IL1R1 | TTGGGTTAAGAGGACAGGGA | TGATTTCTTCTCTGGAGGCTG |
| IL1RN | GCTTGCATCTTGCTGGATTT | CCTCAGAAGACCTCCTGTCCT |
| IL1RAP | ATTAAAGGGAGGGGCAAGAG | CCTCTCAGGTTCCCAAGAAA |
| NOS2 | CGCTACAACATCCTGGAG | ACATTCTGCTTCTGGAAACTA |
| GAPDH | AATGAAGGGGTCATTGATGG | AAGGTGAAGGTCGGAGTCAA |
