# Supplementary material for: C-terminal interleukin 1 alpha (IL-1α) overexpression drives EMT and a vulnerability to ferroptosis in HNSCC
Source: Redox Biol. 2026 Apr 16;93:104172. doi: 10.1016/j.redox.2026.104172 (PMC13122707; doi:10.1016/j.redox.2026.104172)
Supplement: Multimedia component 3 [file mmc3.pptx]

## Slide 1
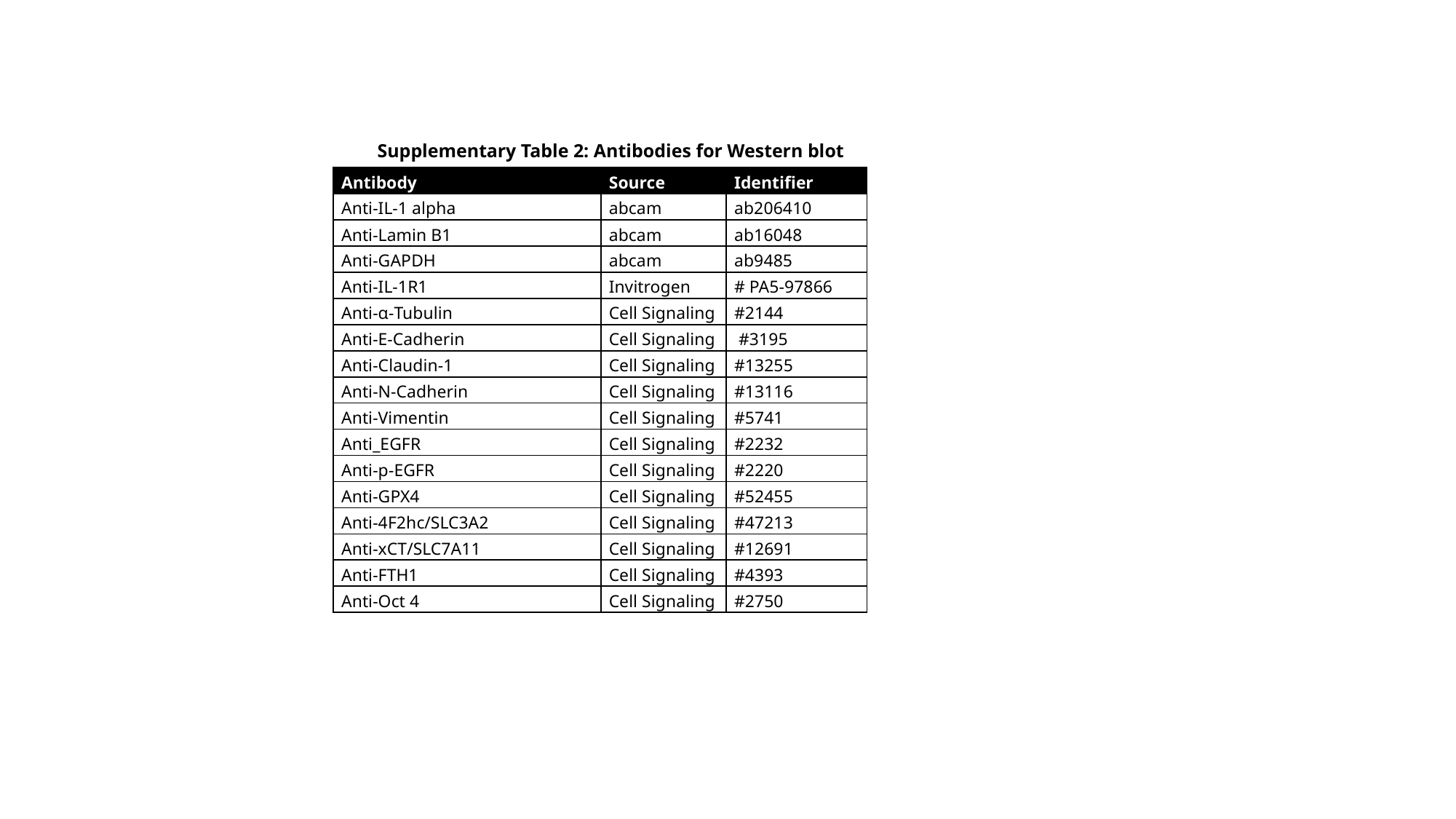

Supplementary Table 2: Antibodies for Western blot
| Antibody | Source | Identifier |
| --- | --- | --- |
| Anti-IL-1 alpha | abcam | ab206410 |
| Anti-Lamin B1 | abcam | ab16048 |
| Anti-GAPDH | abcam | ab9485 |
| Anti-IL-1R1 | Invitrogen | # PA5-97866 |
| Anti-α-Tubulin | Cell Signaling | #2144 |
| Anti-E-Cadherin | Cell Signaling | #3195 |
| Anti-Claudin-1 | Cell Signaling | #13255 |
| Anti-N-Cadherin | Cell Signaling | #13116 |
| Anti-Vimentin | Cell Signaling | #5741 |
| Anti\_EGFR | Cell Signaling | #2232 |
| Anti-p-EGFR | Cell Signaling | #2220 |
| Anti-GPX4 | Cell Signaling | #52455 |
| Anti-4F2hc/SLC3A2 | Cell Signaling | #47213 |
| Anti-xCT/SLC7A11 | Cell Signaling | #12691 |
| Anti-FTH1 | Cell Signaling | #4393 |
| Anti-Oct 4 | Cell Signaling | #2750 |
