# Supplementary material for: C-terminal interleukin 1 alpha (IL-1α) overexpression drives EMT and a vulnerability to ferroptosis in HNSCC
Source: Redox Biol. 2026 Apr 16;93:104172. doi: 10.1016/j.redox.2026.104172 (PMC13122707; doi:10.1016/j.redox.2026.104172)
Supplement: Multimedia component 4 [file mmc4.pptx]

## Slide 1
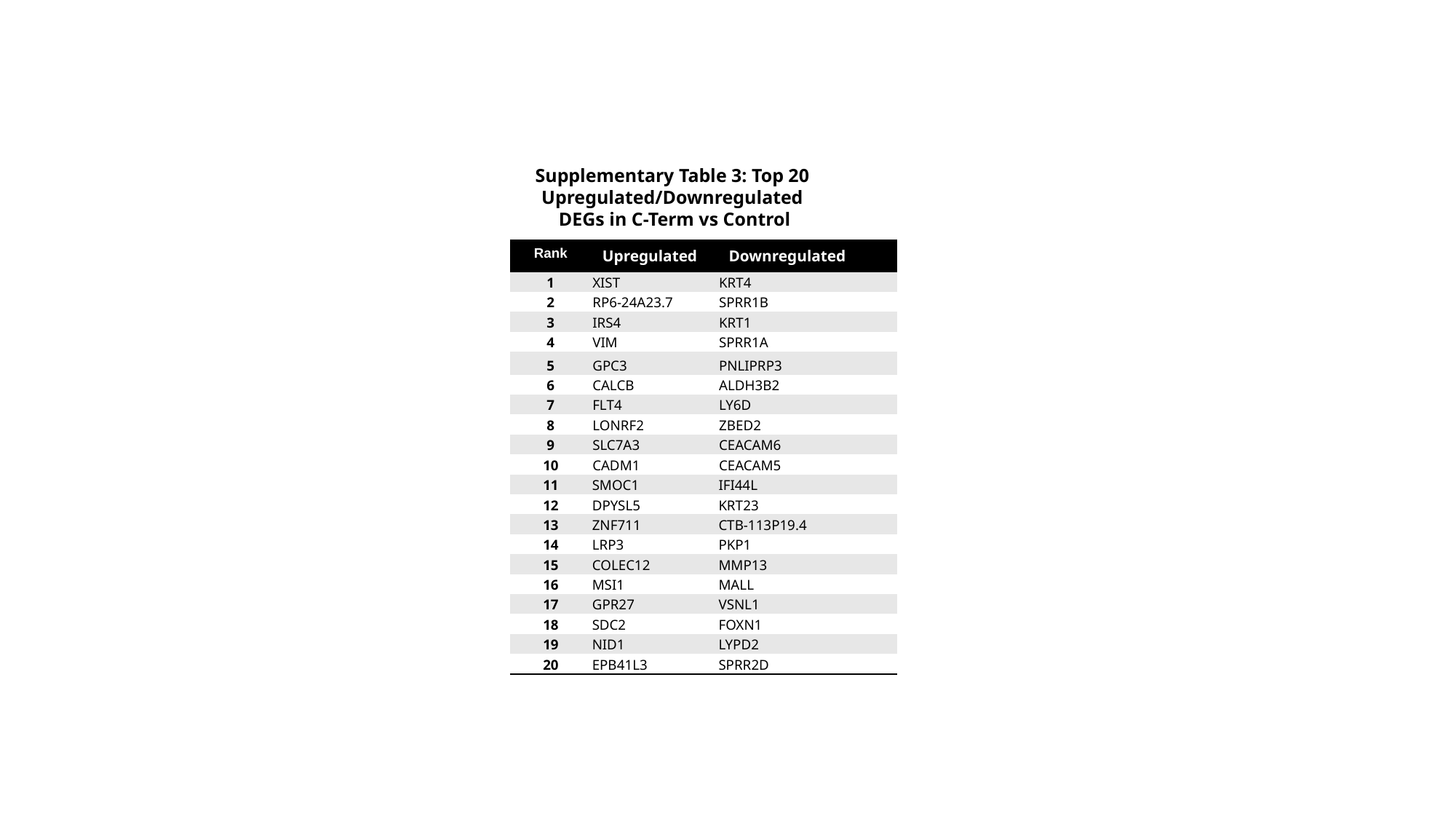

Supplementary Table 3: Top 20
Upregulated/Downregulated
DEGs in C-Term vs Control
| Rank | Upregulated | Downregulated |
| --- | --- | --- |
| 1 | XIST | KRT4 |
| 2 | RP6-24A23.7 | SPRR1B |
| 3 | IRS4 | KRT1 |
| 4 | VIM | SPRR1A |
| 5 | GPC3 | PNLIPRP3 |
| 6 | CALCB | ALDH3B2 |
| 7 | FLT4 | LY6D |
| 8 | LONRF2 | ZBED2 |
| 9 | SLC7A3 | CEACAM6 |
| 10 | CADM1 | CEACAM5 |
| 11 | SMOC1 | IFI44L |
| 12 | DPYSL5 | KRT23 |
| 13 | ZNF711 | CTB-113P19.4 |
| 14 | LRP3 | PKP1 |
| 15 | COLEC12 | MMP13 |
| 16 | MSI1 | MALL |
| 17 | GPR27 | VSNL1 |
| 18 | SDC2 | FOXN1 |
| 19 | NID1 | LYPD2 |
| 20 | EPB41L3 | SPRR2D |
