# Supplementary figures and images for: C-terminal interleukin 1 alpha (IL-1α) overexpression drives EMT and a vulnerability to ferroptosis in HNSCC
Source: Redox Biol. 2026 Apr 16;93:104172. doi: 10.1016/j.redox.2026.104172 (PMC13122707; doi:10.1016/j.redox.2026.104172)

## Slide 1
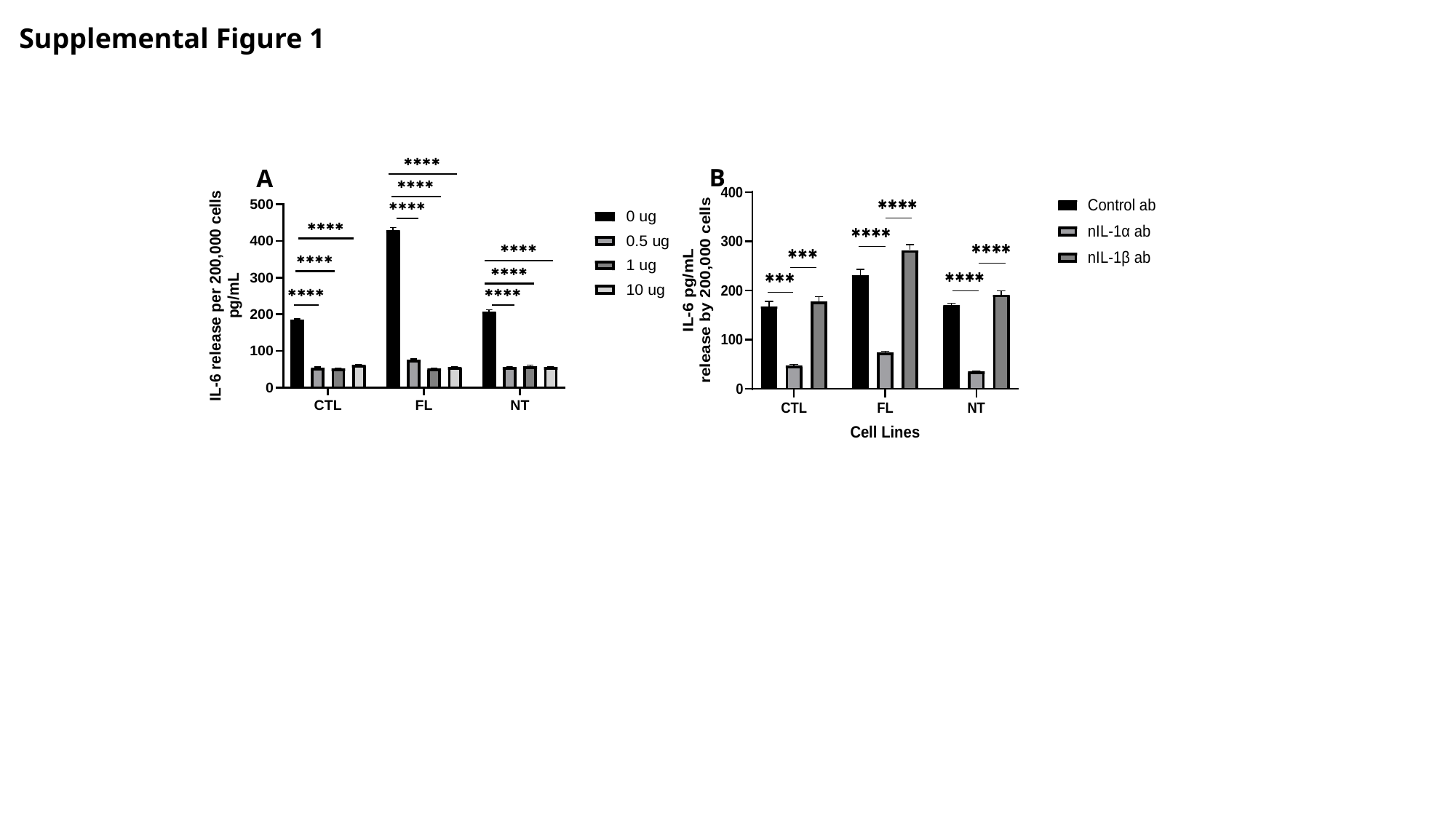

Supplemental Figure 1
B
A

Supplement: Multimedia component 5 [file mmc5.pptx]

## Slide 1
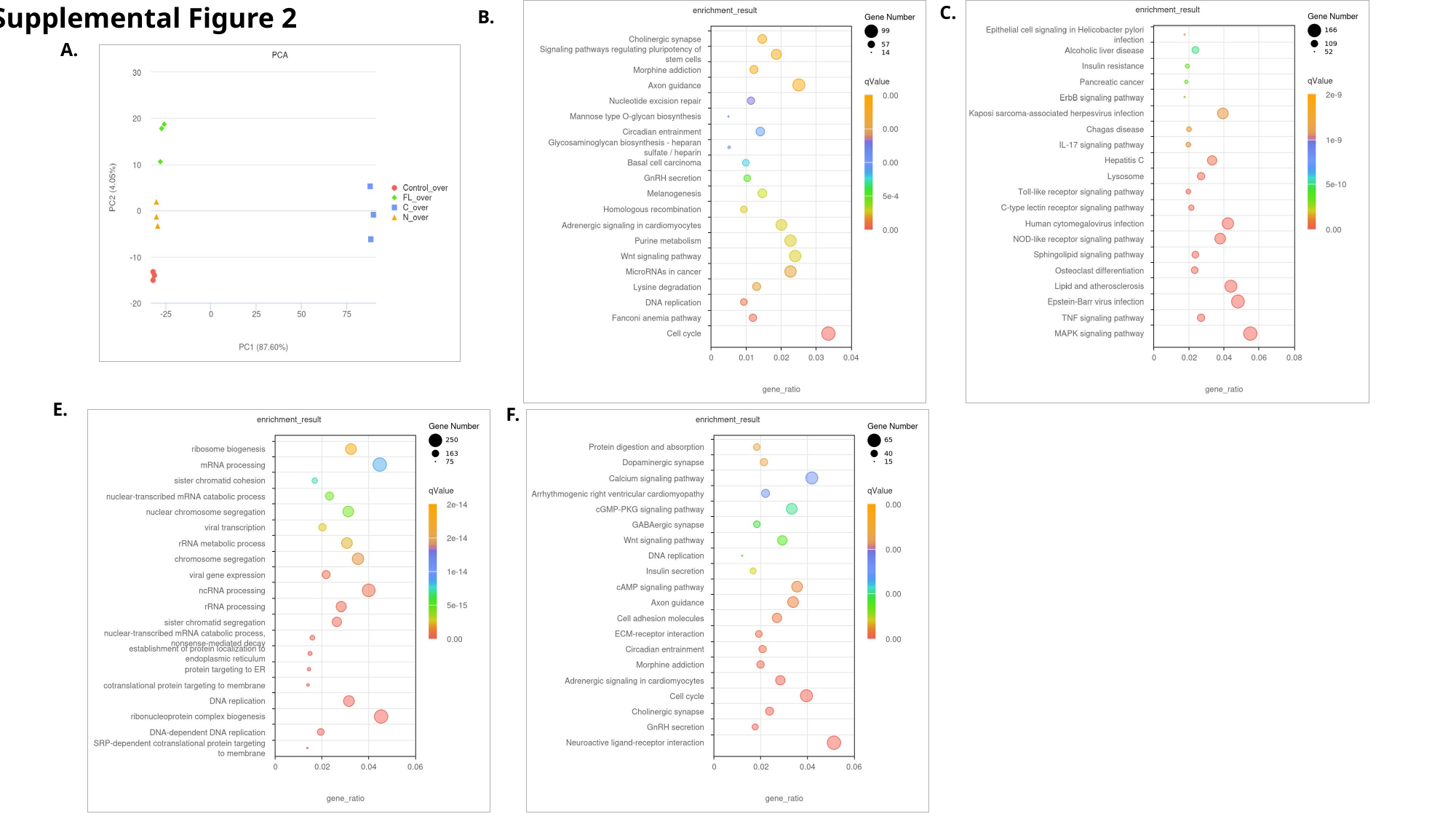

B.
Supplemental Figure 2
C.
A.
E.
F.

Supplement: Multimedia component 6 [file mmc6.pptx]

## Slide 1
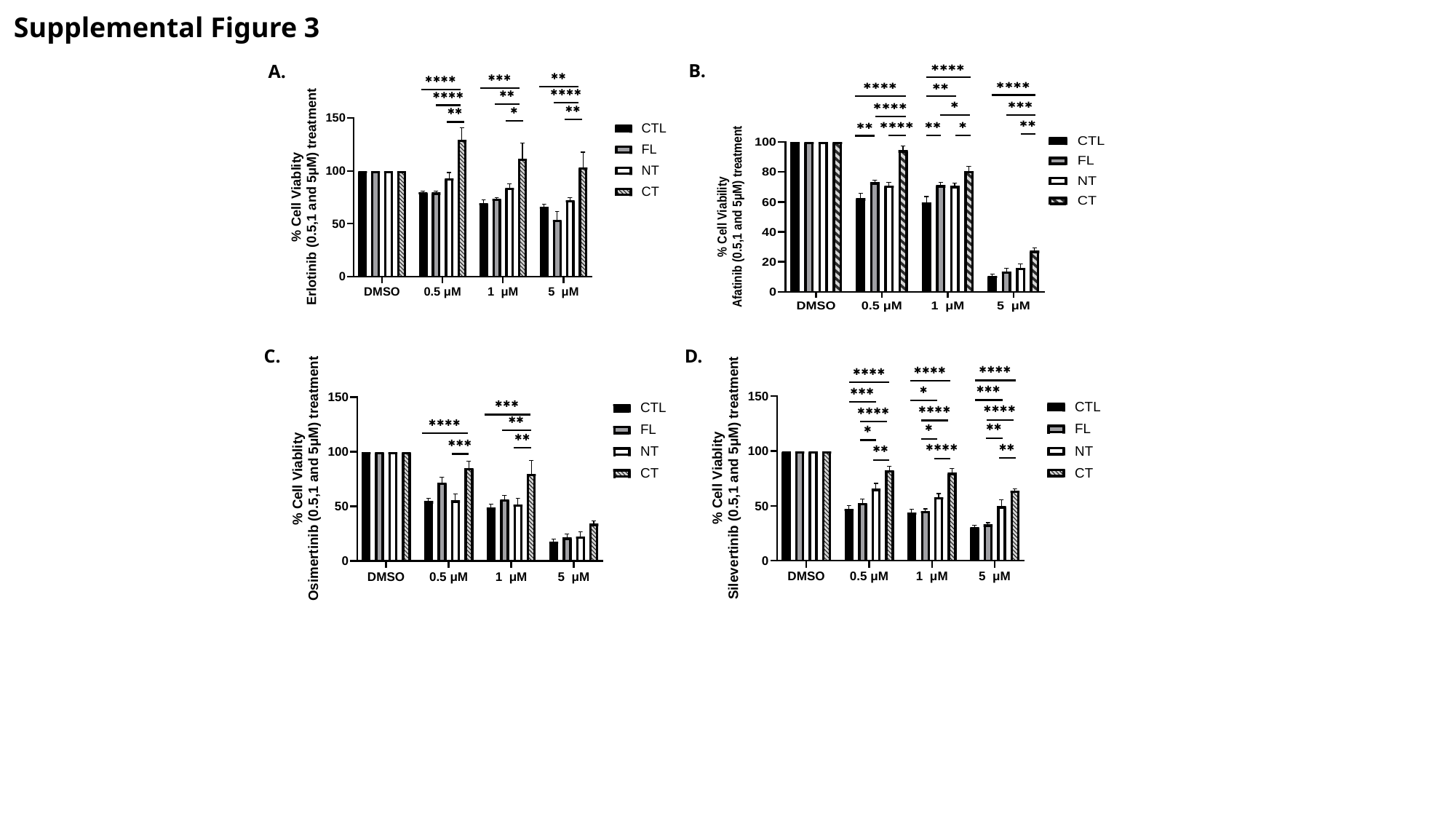

Supplemental Figure 3
B.
A.
D.
C.

Supplement: Multimedia component 7 [file mmc7.pptx]

## Slide 1
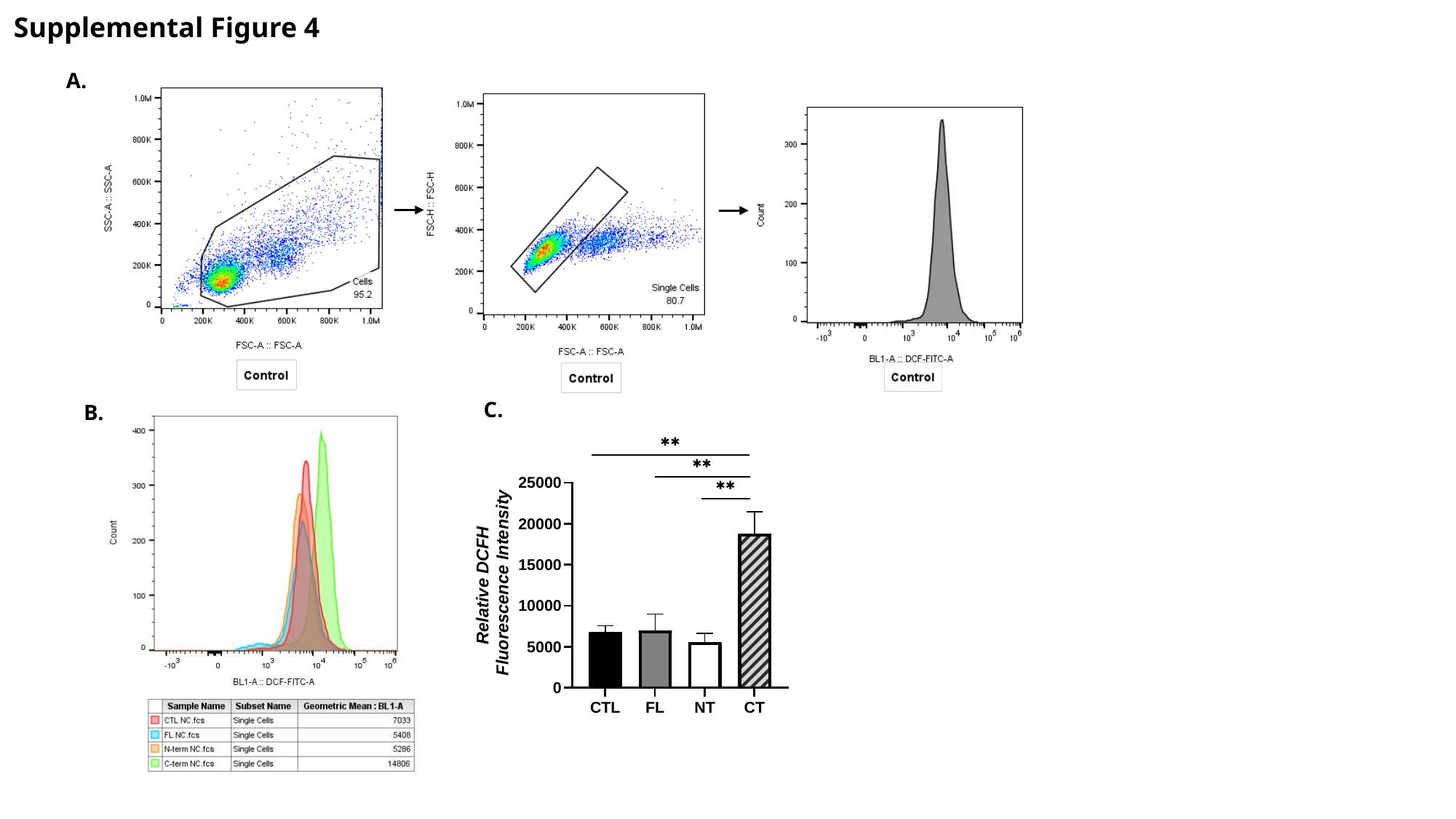

Supplemental Figure 4
A.
C.
B.

Supplement: Multimedia component 8 [file mmc8.pptx]

## Slide 1
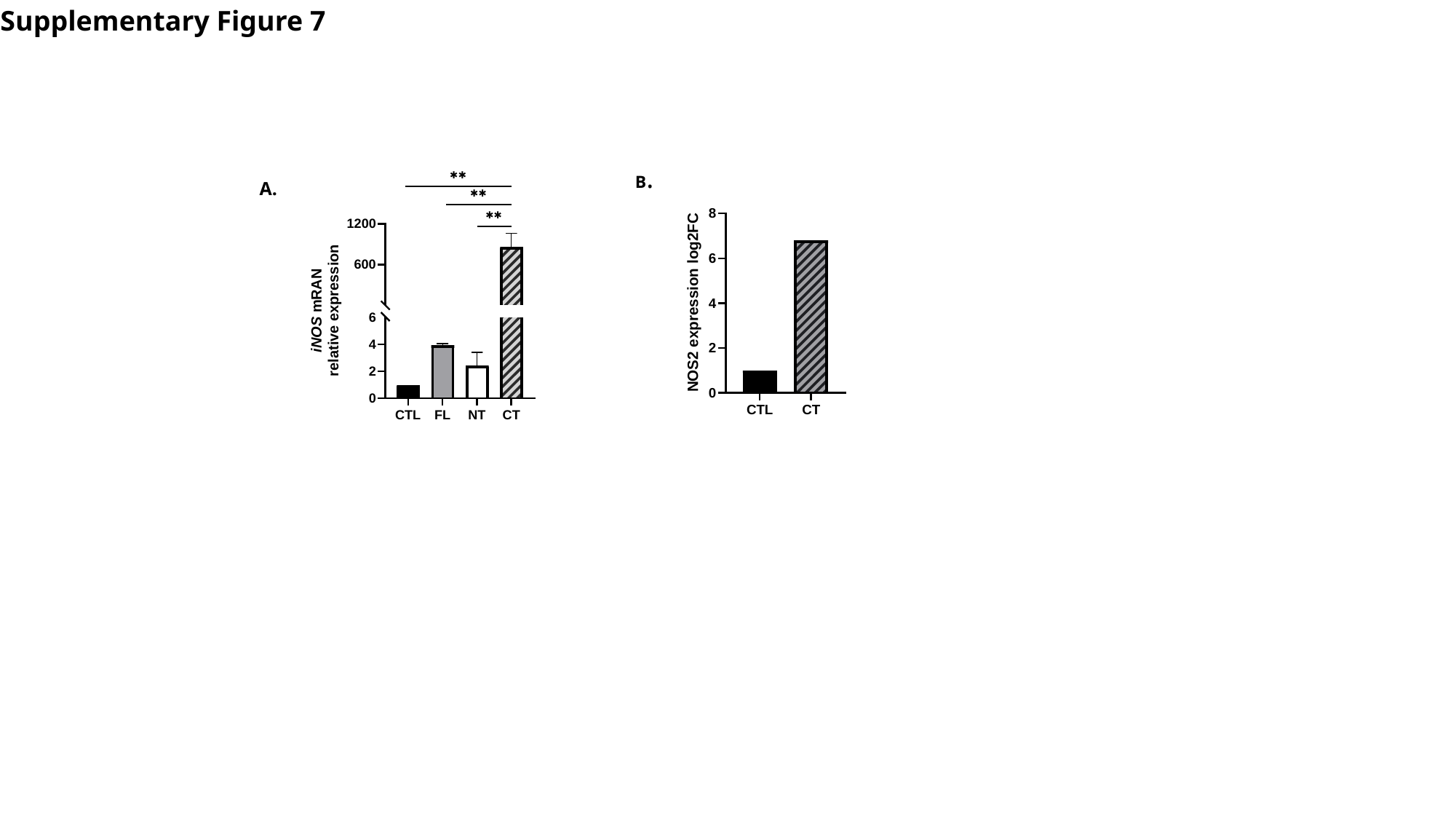

Supplementary Figure 7
B.
A.

Supplement: Multimedia component 11 [file mmc11.pptx]
