# Supplementary material for: C-terminal interleukin 1 alpha (IL-1α) overexpression drives EMT and a vulnerability to ferroptosis in HNSCC
Source: Redox Biol. 2026 Apr 16;93:104172. doi: 10.1016/j.redox.2026.104172 (PMC13122707; doi:10.1016/j.redox.2026.104172)
Supplement: Multimedia component 9 [file mmc9.pptx]

## Slide 1
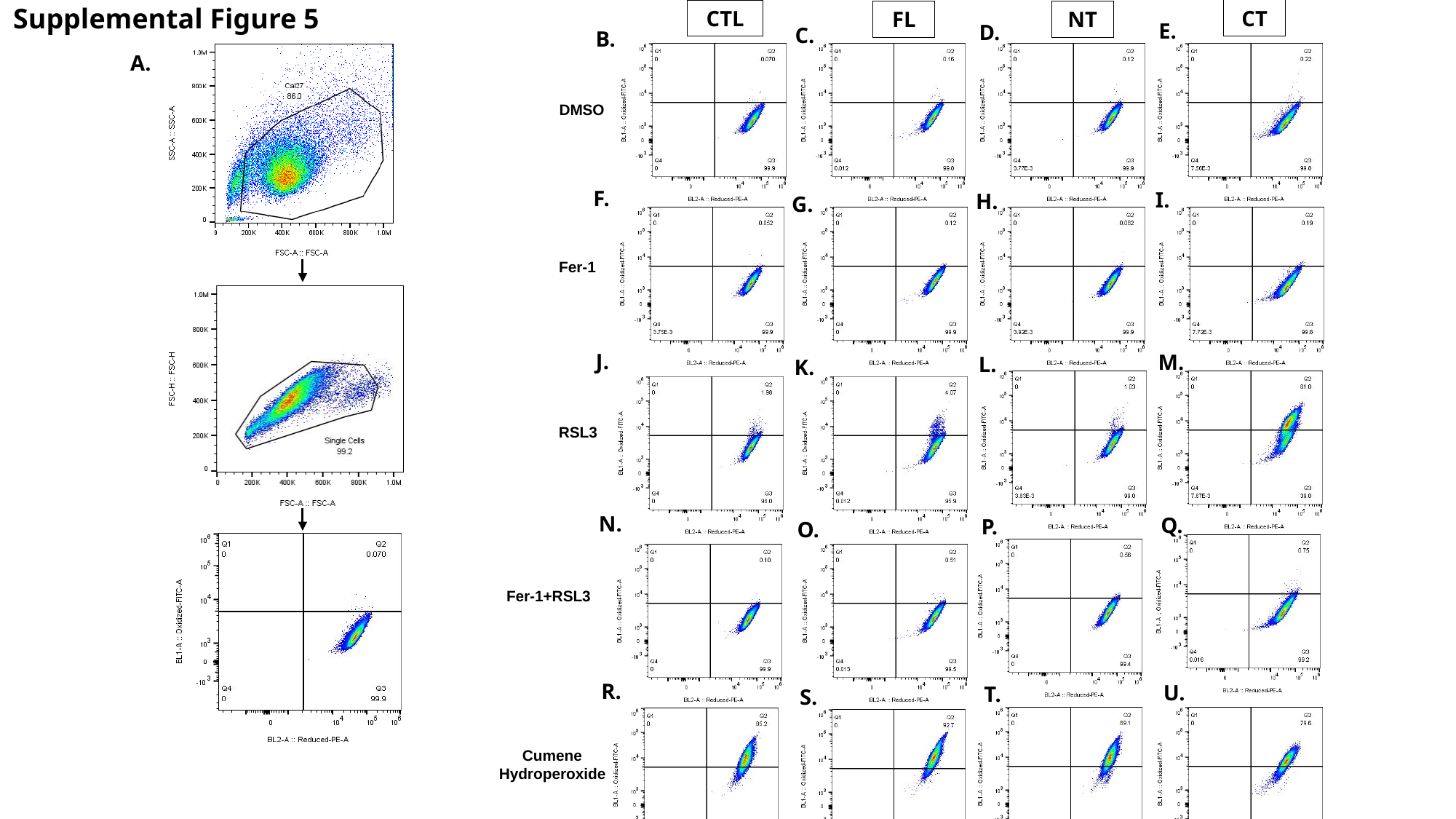

CT
Supplemental Figure 5
CTL
FL
NT
E.
D.
C.
B.
A.
DMSO
F.
I.
H.
G.
Fer-1
J.
M.
L.
K.
RSL3
N.
Q.
P.
O.
Fer-1+RSL3
R.
U.
T.
S.
Cumene
Hydroperoxide
