# Supplementary material for: C-terminal interleukin 1 alpha (IL-1α) overexpression drives EMT and a vulnerability to ferroptosis in HNSCC
Source: Redox Biol. 2026 Apr 16;93:104172. doi: 10.1016/j.redox.2026.104172 (PMC13122707; doi:10.1016/j.redox.2026.104172)
Supplement: Multimedia component 10 [file mmc10.pptx]

## Slide 1
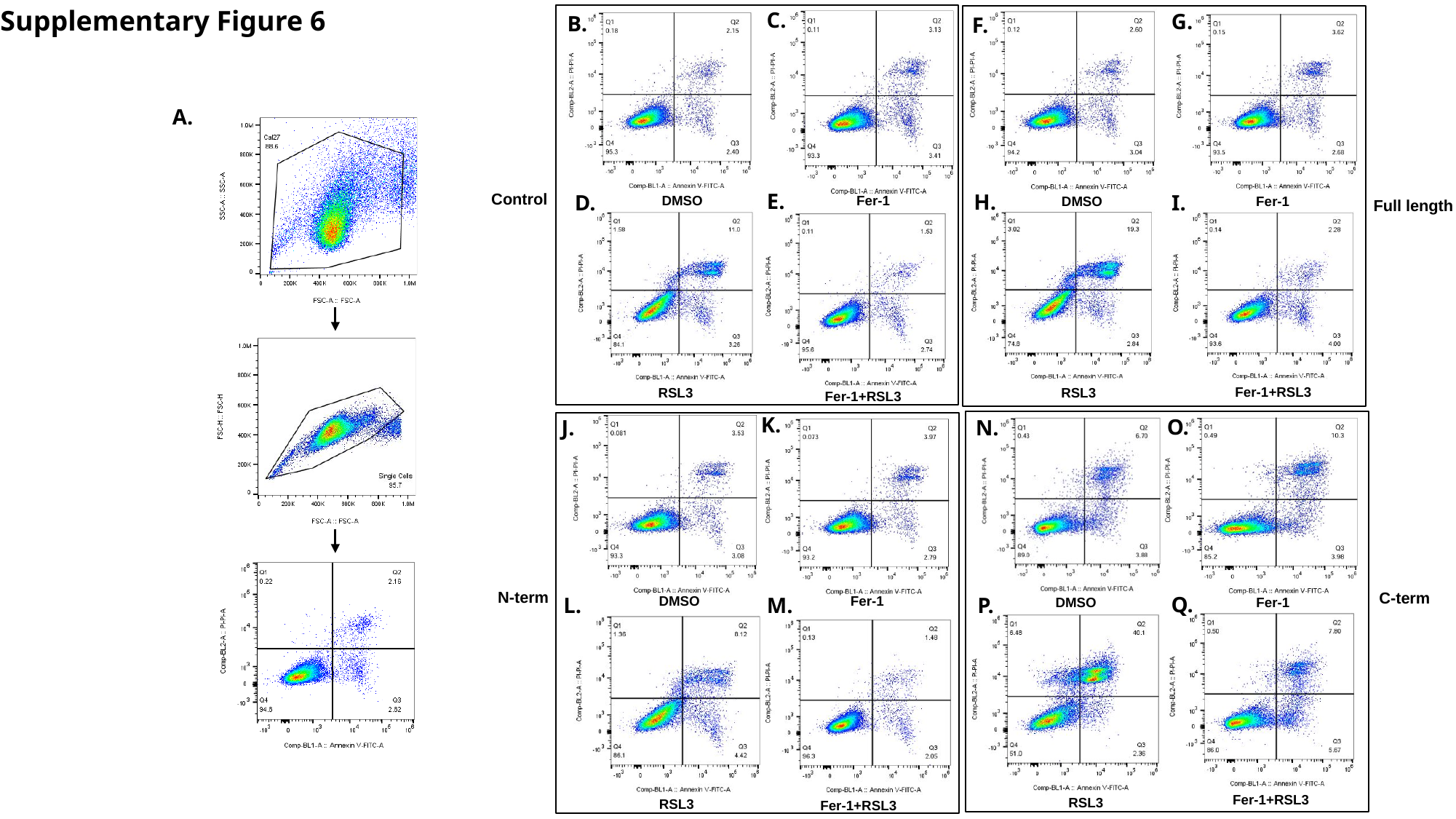

Supplementary Figure 6
C.
G.
B.
F.
A.
E.
Control
H.
I.
D.
DMSO
Fer-1
DMSO
Fer-1
Full length
Fer-1+RSL3
RSL3
RSL3
Fer-1+RSL3
K.
O.
N.
J.
N-term
C-term
Q.
P.
L.
M.
DMSO
Fer-1
DMSO
Fer-1
Fer-1+RSL3
RSL3
RSL3
Fer-1+RSL3
